# Jieduquyuziyin Prescription Suppresses Inflammatory Activity of MRL/lpr Mice and Their Bone Marrow-Derived Macrophages *via* Inhibiting Expression of IRAK1-NF-κB Signaling Pathway

**DOI:** 10.3389/fphar.2020.01049

**Published:** 2020-07-14

**Authors:** Lina Ji, Xuemin Fan, Xiaoli Hou, Danqing Fu, Jie Bao, Aiwen Zhuang, Sixiang Chen, Yongsheng Fan, Rongqun Li

**Affiliations:** ^1^ The First College of Clinical Medicine, Zhejiang Chinese Medical University, Hangzhou, China; ^2^ School of Basic Medical Sciences, Zhejiang Chinese Medical University, Hangzhou, China; ^3^ Academy of Chinese Medical Science, Zhejiang Chinese Medical University, Hangzhou, China; ^4^ Institute of TCM Literature and Information, Zhejiang Academy of Traditional Chinese Medicine, Hangzhou, China; ^5^ The Second College of Clinical Medicine, Zhejiang Chinese Medical University, Hangzhou, China

**Keywords:** Jieduquyuziyin prescription, MRL/lpr mice, bone marrow-derived macrophages, IRAK1-NF-κB signalling, systemic lupus erythematosus, inflammatory activity

## Abstract

Jieduquyuziyin prescription (JP) has been used to treat systemic lupus erythematosus (SLE). Although the effectiveness of JP in the treatment of SLE has been clinically proven, the underlying mechanisms have yet to be completely understood. We observed the therapeutic actions of JP in MRL/lpr mice and their bone marrow-derived macrophages (BMDMs) and the potential mechanism of their inhibition of inflammatory activity. To estimate the effect of JP on suppressing inflammatory activity, BMDMs of MRL/lpr and MRL/MP mice were treated with JP-treated serum, and MRL/lpr mice were treated by JP for 8 weeks. Among them, JP and its treated serum were subjected to quality control, and BMDMs were separated and identified. The results showed that in the JP group of BMDMs stimulated by Lipopolysaccharide (LPS) in MRL/lpr mice, the secretion of interleukin-6 (IL-6) and tumor necrosis factor-α (TNF-α) reduced, and the expressions of Interleukin-1 receptor-associated kinase 1 (IRAK1) and its downstream nuclear factor κB (NF-κB) pathway decreased. Meanwhile, the alleviation of renal pathological damage, the decrease of urinary protein and serum anti-dsDNA contents, the inhibition of TNF-α level, and then the suppression of the IRAK1-NF-κB inflammatory signaling in the spleen and kidney, confirmed that the therapeutic effect of JP. These results demonstrated that JP could inhibit the inflammatory activity of MRL/lpr mice and their BMDMs by suppressing the activation of IRAK1-NF-κB signaling and was supposed to be a good choice for the treatment of SLE.

## Introduction

Systemic lupus erythematosus (SLE) is the most severe type of lupus erythematosus, and it is an autoimmune-mediated chronic diffuse connective tissue disease involving multiple organs in the body, which is characterized by immune inflammation (Muñoz et al., 2011; Casciato et al., 2018). The current treatments for SLE include glucocorticoids and immunosuppressant agents. Although these conventional treatments can relieve certain symptoms and temporarily prevent disease progression, they have limited therapeutic effects (Li et al., 2017). Additionally, to date, the biological agent belimumab is the first drug approved for the treatment of SLE, but scores of immune indicators remain under study (Murphy et al., 2013). In China, some clinical Chinese medicine formulas for SLE which include Jieduquyuziyin prescription (JP) are generally accepted by many people because of the due effect and minimal adverse reactions (Fan et al., 2005; Fan, 2019).

JP has been exclusively and validly applied in the treatment of SLE for more than a decade in the Chinese Traditional Medicine Hospital of Zhejiang Province, China. The whole prescription consists of ten traditional Chinese herbs (Ji et al., 2019b). Many molecular biological researches and clinical data proved that it could adjust immunity, control inflammation, lessen the by-effects of western medicine, as well as reduce the occurrence of infections (Wen et al., 2007; Shui et al., 2015; Li et al., 2018b). Simultaneously, JP had stable and reliable quality, and the effective ingredients in JP were identified using rapid resolution liquid chromatography tandem triple quadrupled mass spectrometer (RRLC-QqQ-MS/MS) (Hu et al., 2013; Ding et al., 2014). Furthermore, treatment of MRL/lymphoproliferation spontaneous mutation (lpr) mice with JP active ingredients has demonstrated protective effects of JP on the pathology of SLE (Ji et al., 2018). Although JP has been proved to be efficacious in the treatment of SLE, little is known about the exact mechanism and target of drug, so it is necessary to find new drug targets for SLE.

Notably, inflammatory signals disorder in disease is the cause of SLE (Li et al., 2017). Toll-like receptors (TLRs) belong to the membrane proteins family that are involved in recognition of microorganisms and induction of inflammatory procedures. Concurrently, it is also an efficacious therapeutic target for autoimmune diseases, including SLE (Summers et al., 2010; Sahu et al., 2014). Interleukin-1 receptor-associated kinase 1 (IRAK1) is a critical molecule in the TLR4 and its myeloid differentiation primary response 88 (MyD88) pathway. Then IRAK1 will be phosphorylated to form a complex with tumor necrosis factor (TNF) receptor associated factor 6 (TRAF6). The nuclear factor κB inhibitor (IκB) is subsequently activated by the complex and induces the activation of nuclear factor κB (NF-κB). Ultimately, NF-κB induces large production of inflammatory cytokines (Flannery and Bowie, 2010; Heiseke et al., 2015; Zhou et al., 2019).

Researches confirmed that IRAK1 gene polymorphisms played a key role in the pathogenesis of lupus (Jacob et al., 2009; Sánchez et al., 2011; Kaufman et al., 2013; Doudar et al., 2019). Additionally, researches have shown that specific inhibition of IRAK1 kinase may have therapeutic potential to prevent SLE (Nanda et al., 2016). The NF-κB signaling disorder acts a pivotal part in various inflammatory and autoimmune diseases, and is also an important transcription factor for the development of SLE disease (Marshak-Rothstein, 2006; Fu et al., 2019). Endotoxin, a component of Gram-negative bacteria, activates many major cellular effects which are crucial in the inflammatory response (Chen et al., 2015). Lipopolysaccharide (LPS) activates TLR4 and causes a cascade of intracellular inflammatory signals, including inhibitor of nuclear factor kappa-B kinase (IKK)/IκB/NF-κB (Wang et al., 2014; Ding et al., 2016; Wang et al., 2017). The above demonstrates that IRAK1-NF-κB is a significant determinant in the pathogenesis of SLE. However, few functional studies on these two signaling pathways in SLE have been published.

Prior to this, we mainly studied IRAK1 and NF-κB related pathways in mouse peritoneal macrophages (Ji et al., 2018). The effects of JP on IRAK1 and its downstream NF-κB activation were analyzed by *in vivo* (animal) and *in vitro* (cell) experiments, and its regulatory effect on inflammatory responses was explored. We hypothesized that JP’s ability of improving inflammatory activity in SLE based on reducing IRAK1-NF-κB signaling in MRL/lpr mice and their bone marrow-derived macrophages (BMDMs).

## Materials and Methods

### Drugs

JP was composed of 10 herbs (**Table 1**) which were mixed according to the mass ratio 5:4:4:5:4:5:4:3:3:2 (Ji et al., 2019a). Among them, *Rehmannia glutinosa* (Gaertn.) DC (batch no. 191101), *Trionyx sinensis* Wiegmann (batch no. 191001), *Artemisia annua* L. (batch no. 191001), *Scleromitrion diffusum* (Willd.) R.J.Wang (syn. *Hedyotis diffusa* Willd.) (batch no. 191101), *Paeonia anomala* subsp. *veitchii* (Lynch) D.Y.Hong & K.Y.Pan (syn. *Paeonia veitchii* Lynch) (batch no. 191001), *Centella asiatica* (L.) Urb. (batch no. 191101), *Paeonia x suffruticosa* Andr. (batch no. 191101), *Citrus medica* L. (batch no. 191001), *Actaea cimicifuga* L. (syn. *Cimicifuga foetida* L.) (batch no. 191101), and *Glycyrrhiza uralensis* Fisch. ex DC. (batch no. 191001) were purchased from Zhejiang Chinese Medical University Medical Pieces., LTD (Hangzhou, China). All the above plant samples were stored as voucher specimens in the Public Platform of Medical Research Center, Academy of Chinese Medical Science, Zhejiang Chinese Medical University (Hangzhou, China), and the voucher numbers of the plant samples corresponded to No. GDH-2019-0010, No. ZBJ-2019-0010, No. QH-2019-0010, No. BHSSC-2019-0010, No. CS-2019-0010, No. JXC-2019-0010, No. DP-2019-0010, No. FS-2019-0010, No. SM-2019-0010, and No. SGC-2019-0010 respectively. The total 117 g of mixed medicine was boiled with 500 ml of water for 30 min according to the references (Li et al., 2018a; Li et al., 2018b). This crude drug decoction was filtrated and concentrated to 1.56 g/ml, then preserved at 4°C and re-warmed before administration.

**Table 1:** The compositions of Jieduquyuziyin prescription (JP).

Chinese name	Latin name	Scientific name	Weight (g)	Parts used
Gan Di Huang	Rehmanniae radix	*Rehmannia glutinosa* (Gaertn.) DC.	15	Root Tuber
Zhi Bie Jia	Trionycis carapax	*Trionycis carapax* (Wiegmann)	12	Tergum
Qing Hao	Artemisiae annuae herba	*Artemisia annua* L.	12	Herb
Bai Hua She She Cao	Herba hedyotidis diffusae	*Scleromitrion diffusum* (Willd.) R.J.Wang (syn. *Hedyotis diffusa* Willd.)	15	Herb
Chi Shao	Paeoniaeradix rubra	*Paeonia anomala* subsp. veitchii (Lynch) D.Y.Hong & K.Y.Pan (syn. *Paeonia veitchii* Lynch)	15	Root
Ji Xue Cao	Centellae herba	*Centella asiatica* (L.)	12	Herb
Dan Pi	Moutan cortex	*Paeonia × suffruticosa* Andrews	12	Root
Fo Shou	Citri sarcodactylis fructus	*Citrus medica* L.	9	Fruit
Sheng Ma	Cimicifugae rhizoma	*Actaea cimicifuga* L. (syn. *Cimicifuga foetida* L.)	9	Rhizome
Sheng Gan Cao	Glycyrrhizae radix et rhizoma	*Glycyrrhiza uralensis* Fisch. ex DC.	6	Root

### High-Performance Liquid Chromatography (HPLC) for Identification and Content Determination

Gallic acid (HPLC ≥98%), Paeoniflorin (HPLC ≥98%), Ferulic acid (HPLC ≥98%), Isoferulic acid (HPLC ≥98%) were prepared (Yuanye, Shanghai, China). The HPLC system (Waters, MA, United States) comprised a Waters 2695 separation unit, a 2996 diode array detector, and a 2420 evaporative light scattering detector. In addition, the temperature was settled at 30°C and the chromatographic column used was a Hypersil BDS C18 column (250 × 4.6 mm, 5 μm) (Elite, Dalian, China). The UV spectra were 271 nm, and the mobile phases were a mixture of acetonitrile (A) and 0.2% acetic acid (B). The flow rate was 1.0 ml/min and the gradient program was used: 0–7 min, 7% A; 7–8 min, 7–19% A; 8–11 min, 19% A; 11–20 min, 19–33.68% A; 20–25 min, 33.68–7% A; 25–30 min, 7% A. The compounds in JP were qualified using standard calibration curves.

### Preparation of JP Serum

Clean Sprague-Dawley male rats weighing 180–220 g were used in the experiment, provided by Animal Experiment Center of Zhejiang Chinese Medical University. These rats were randomly divided into a control group (n = 10) and a JP-treated group (n = 10). On the basis of dose conversion from human to rat according to clinical application, the dosage of JP was 1 ml/100g (Li et al., 2018b). Treatment was chosen for 5 days, then obtained rat blood after the last drug administration. After standing at room temperature for 2 h, the collected blood was centrifuged at 3,000 r/min for 15 min to separate the serum. And then the serum was inactivated at 56°C for 30 min, filtered and stored in −80°C refrigerator (Hu et al., 2018).

### Determination by Liquid Chromatography-Mass Spectrometry

Chemical constituents of JP were identified by liquid chromatography-mass spectrometry (LC-MS) which was also used to control the quality. The serum of the blank group and the JP group were placed in a centrifuge tube, and triple the amount of methanol was added. The supernatant was vortexed and centrifuged, dried with N_2_, dissolved in methanol, and finally passed through a microporous membrane (Shimadzu, Kyoto, Japan).

Chromatographic conditions: InertSustain C18 (5 μm, 4.6 × 150 mm) column (Shimadzu, Kyoto, Japan), the column temperature was 40°C. Mobile phase A is acetonitrile and B is pure water containing 0.1% formic acid, and the gradient elution procedure for acetonitrile is 15–50% (0–9 min), 50–90% A (9–11 min), 90% (11–17 min), 90–15% (17–19 min), 15% (19–20 min) gradient elution. Besides, the flow rate was 0.5 ml/min, and the injection volume was 5 ul.

Mass spectrometry conditions referred to our previous experiments. And the quantitative ion pairing of paeoniflorin was 479.15 m/z to 121.05 m/z and ferulic acid was 193.05 m/z to 134.10 m/z. The full scan mode was adopted, and the scanning range was m/z 100 to 1,000 Da (Li et al., 2018b).

### *In Vitro*

#### Culture and Maintenance of BMDMs

For mouse BMDMs preparation, cells were flushed from femur and tibia bones of the above mice strains between 14 and 16 weeks of age (WKE et al., 2017). The cells were washed with pre-chilled Phosphate buffered saline (PBS) and cultured in cell culture dishes (ø 100mm, Corning, NYC, United States). And the cells grew in Dulbecco’s modified Eagle’s medium (Gibco, CA, United States) containing L-glutamine, 15% fetal bovine serum (FBS, Gemini, CA, United States), and 25 ng/ml macrophage colony-stimulating factor (M-CSF) (Pepro Tech, NJ, United States) in complete media. On day 3, suspended cells were removed by fluid exchange, and then the fresh medium was added. During the period, the medium could be changed according to the state of the cells. At the end of the sixth day, adhered BMDMs were harvested and used for experiments (Wex et al., 2015).

#### Flow Cytometric Assay

On day 6, the supernatant was drawn off, and the BMDMs were washed PBS and harvested with trypsin digestion. The BMDMs were washed, then blocked by the antibody with anti-mouse classification determinant (CD) 16/32 receptor (Biolegend, CA, United States) and incubated. Then, the BMDMs were incubated with CD11b-APC and F4/80-FITC (all from eBioScience, CA, United States) for 30 min. Detected cells by flow cytometry (BD, CA, United States) as soon as possible after washing with pre-chilled PBS.

#### Cell Viability Analysis

BMDMs were incubated in a 96-well plate, and drug intervention was performed when the cell state was stable. Subsequent treatment with JP-treated serum at concentrations of 2.5, 5, 7.5, 10, 15, 20, and 30% (v/v). BMDMs viability was tested by Cell Counting Kit-8 (CCK8) (Beyotime, Shanghai, China). Then added the CCK8 reagent according to the instructions before detection, and finally measured the absorbance using spectrophotometry.

### *In Vivo*

#### Mice and Treatments

Specific pathogen free (SPF) state 6–8 week old female MRL/lpr and MRL/Mp mice were selected and purchased from Slac Laboratory Animal Co., Ltd. (Shanghai, China). These animals were maintained in a barrier environment and subjected to a 12-h light/dark cycle every day. The environment of the mice was set at a constant temperature of 25°C, relative humidity 40–60%, and provided with free access to a standard diet.

Twenty MRL/lpr mice were randomly distributed into two groups: mice treated with diluted water (Model group) and mice treated with JP (JP group). Mice of the JP-treated group were administered JP [18 ml/kg body weight (bw) per day, i.g.] (Shui et al., 2015). And 10 MRL/Mp mice were treated with diluted water as the control group. The drug and water were administered into the animals by intragastric administration. The treatment time before the mice are sacrificed is from the 7th week, and the gavage time is 8 weeks. The mouse urine was collected for 24 h proteinuria before sacrifice, the mouse serum was collected from the orbital venous plexus before sacrifice, and the mouse spleen and kidney tissue samples were obtained immediately after the sacrifice. All the above animal studies were approved by the Animal Experiment Ethics Committee of Zhejiang University of Traditional Chinese Medicine.

#### Hematoxylin and Eosin Staining

Mouse kidney samples were preserved in 4% paraformaldehyde, made into paraffin blocks, then sliced at a thickness of 4 μm, dewaxed and dehydrated sequentially, and stained with hematoxylin and eosin (H&E). Ultimately, observed and performed pathological analysis under a light microscope (Motic, Xiamen, China).

#### Enzyme-Linked Immunosorbent Assay (ELISA)

BMDMs were incubated in culture plates and treated with 1 μg/ml LPS either alone or with 2.5% (v/v) concentration of JP-treated serum. The concentrations of TNF-α and interleukin (IL)-6 in the supernatant of BMDMs were measured with enzyme-linked immunosorbent assay (ELISA) kits (NOVUS biologicals, CO, USA).

The urine of the mice was centrifuged at 12,000 r/m for 15 min to collect the supernatant, and then the urine protein concentration in the urine was measured according to the LBIS Mouse Albumin ELISA Kit (FUJIFILM, Gunma, Japan). Each mouse venous blood was allowed to stand for 30 min, and the serum was collected by centrifugation. Afterwards, the concentration of anti-dsDNA in the serum was measured with the LBIS Mouse anti-dsDNA ELISA Kit (FUJIFILM, Gunma, Japan). The procedure was strictly in accordance with the kit instructions and the required indicators were measured in a microplate reader (PerkinElmer, EnSpire, MA, United States).

### Quantitative Real-Time Polymerase Chain Reaction Analysis

Extraction of total RNA from BMDMs, spleen, and kidney tissues was used by RNAiso Plus (TAKARA, Dalian, China), and then was transformed into complementary DNA using the TAKARA Reverse Transcription System Kit (TAKARA, Dalian, China). The Roche LightCycler 96 SW1.1 instrument (Roche, Basel, Switzerland) were used for quantitative real-time polymerase chain reaction (Rt-qPCR). The results were analyzed using 2^^(-ΔΔCt)^ values. GAPDH (Sangon Biotech, Shanghai, China) was used as a control. **Table 2** showed the nucleotide sequences of the required primers in the experiment.

**Table 2:** The primer sequence for RT-qPCR.

Gene	Sequence (5’-3’)	
	Forward primer	Reverse primer
IRAK1	GGTCCCTGTCTCTTCCCTTC	GAGGAAGGAATTCAGCCTTTG
NF-κB	GCCGTGGAGTACGACAA	CGGTTTCCCATTTAGTATGT
TNF-α	ACCAGACACCTCAGGGCTAA	TGTTGGGGAGAAGGAGAATG

### Western Blot Analysis

Extractions of total protein from different groups of BMDMs, spleen, and kidney tissues were used by Qproteome Mammalian Protein Prep Kit (Qiagen, Dusseldorf, Germany). The protein concentrations were detected by BCA kit (Biosharp, Hefei, China). The isolated equivalent amount of protein polyvinylidene fluoride (PVDF) membranes were incubated overnight after added IRAK1 (abcam, ab238, 1:1,000), p-IRAK1 (T209) (abcam, ab218130, 1:500), NF-κB (abcam, ab32536, 1:1,000), p-NF-κB (S536) (abcam, ab86299, 1:2,000), IκBα (abcam, ab32518, 1:1,000), p-IκBα (S36) (abcam, ab133462, 1:10,000), and α-tubulin (CST, sc2144, 1:500) individually. The membranes were then incubated with anti-rabbit IgG (1:1,000), anti-mouse IgG (1:1,000) for 1 h, and chemically developed the membranes with ECL Substrate (Bio-Rad, CA, United States). The signals were quantified by an imager (Proteinsimple, CA, United States), and Image J software was used to quantify protein bands as a ratio to α-tubulin.

### Statistical Analysis

All data in the text were expressed as mean ± standard deviation. The *t*-test and one-way (ANOVA) analysis of variance were used to analyze the significance of the differences between the groups. *P* < 0.05 was a statistically significant difference.

## Results

### Quantitative Analysis of the Chemical Constituents of JP and JP-Treated Serum

**Figure 1:**
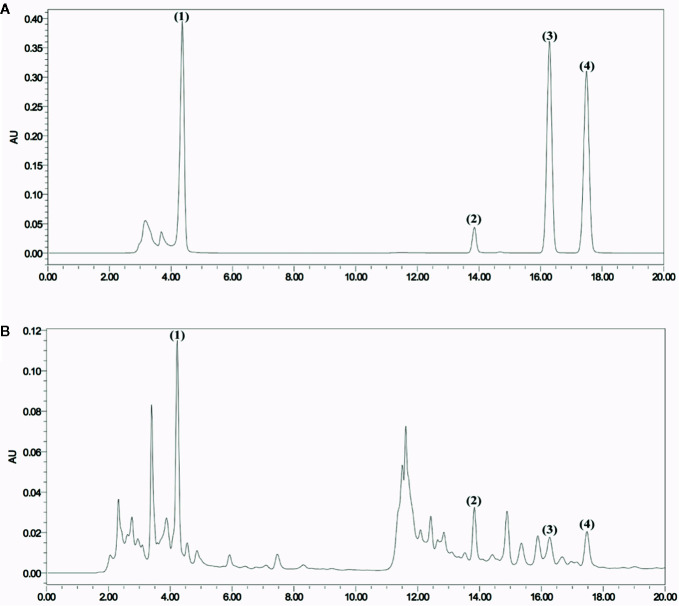
HPLC chromatogram of JP at 271 nm. Mixed reference standard substance **(A)** and JP **(B)**. Identification of main compounds in JP as following: Gallic acid (1), Paeoniflorin (2), Ferulic acid (3), and Isoferulic acid (4).

The chromatogram of the mixed standard and JP were shown in **Figure 1**. The retention times of gallic acid, paeoniflorin, ferulic acid, and isoferulic acid were 4.212, 13.826, 16.280, and 17.492 mins, respectively. Through the calculation of the regression curve, the concentrations of these four components in JP were 11.34, 88.02, 3.02, and 2.57 μg/ml, separately.

**Figure 2:**
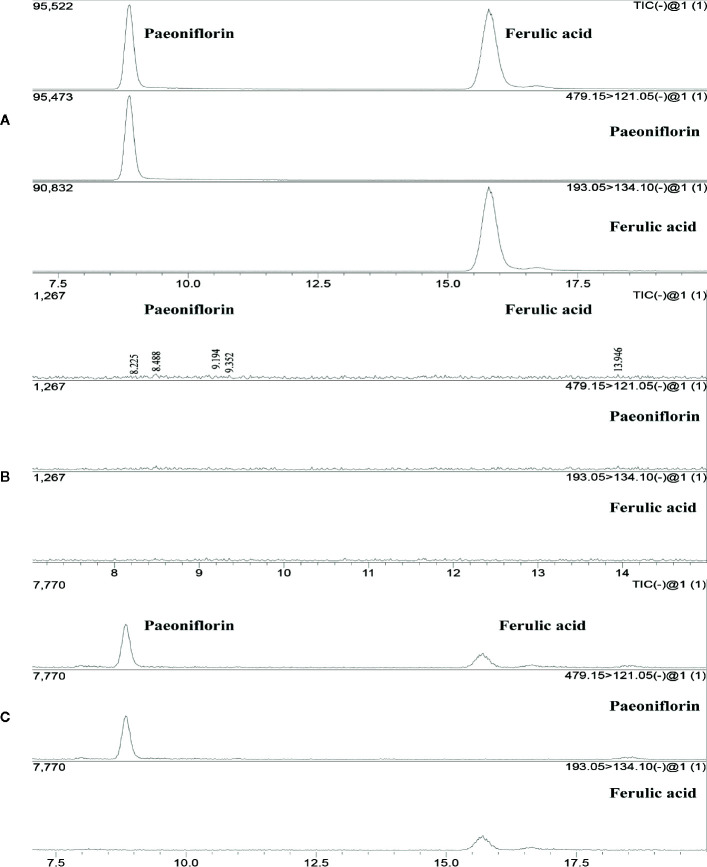
The total ion maps of ferulic acid and paeoniflorin in serum and mixed standards were determined by UPLC-MS/MS. The standard **(A)**, Blank serum group **(B)**, and JP-treated serum group **(C)**.

The experimental results showed an ion chromatogram of JP-treated serum, blank serum, and reference substance in **Figure 2**. The retention times of paeoniflorin and ferulic acid were 8.839 and 15.782 mins. The mass concentration of paeoniflorin in the JP-treated serum was (2.539 ± 0.656) ng/ml, and ferulic acid was (0.350 ± 0.203) ng/ml. However, under the same conditions, the above components were not detected in blank serum.

### The Purity of BMDMs Identified by Flow Cytometry

**Figure 3:**
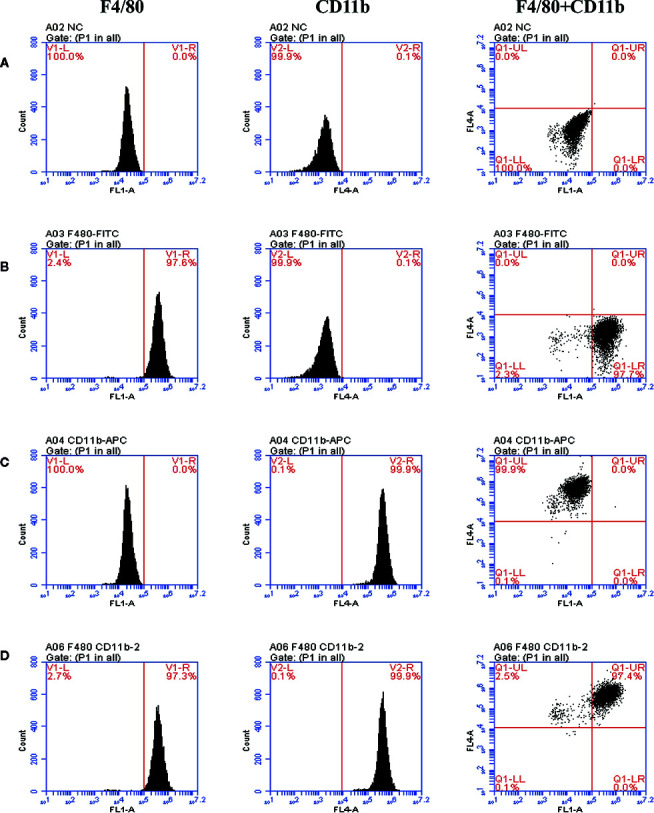
The purity of BMDMs identified by flow cytometry and staining with anti-F4/80 antibody (FITC) and anti-CD11b antibody (APC). The representative scatter diagrams of each group are shown: Control group **(A)**, Anti-F4/80 antibody only group **(B)**, Anti-CD11b antibody only group **(C)**, and Anti-F4/80 antibody and Anti-CD11b antibody group **(D)**.

BMDMs induced on the 7th day of differentiation were induced. The results demonstrated that the number of F4/80 positive cells representing macrophages in macrophage colony-stimulating factor (M-CSF)-induced differentiation of macrophages was 97.3%, and the number of CD11b positive cells was 99.9% (**Figure 3**).

### Effect of JP-Treated Serum on Proliferation of BMDMs

**Figure 4:**
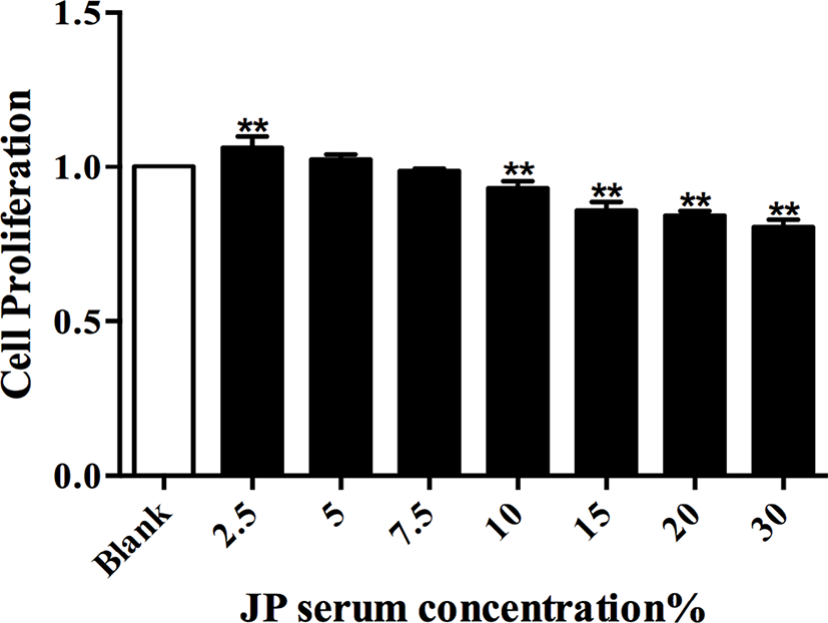
The effect of JP-treated serum for 24 h on the proliferation of BMDMs. Data are shown as the mean ± SD (n = 5) of one representative experiment. **P < 0.01 *versus* Blank group.

The CCK8 detection method was used to explore the effect of JP-treated serum at different concentrations in BMDMs, and then to evaluate the cell viability under the action of JP. Compared to the control group, JP treatment with 2.5% (v/v) concentration had the most remarkable effect on the proliferation of BMDMs (**Figure 4**). Similarly, the concentration of cells intervened with blank serum in subsequent experiments was also 2.5% (v/v).

### JP Relieves Renal Tissue Damage in MRL/lpr Mice

**Figure 5:**
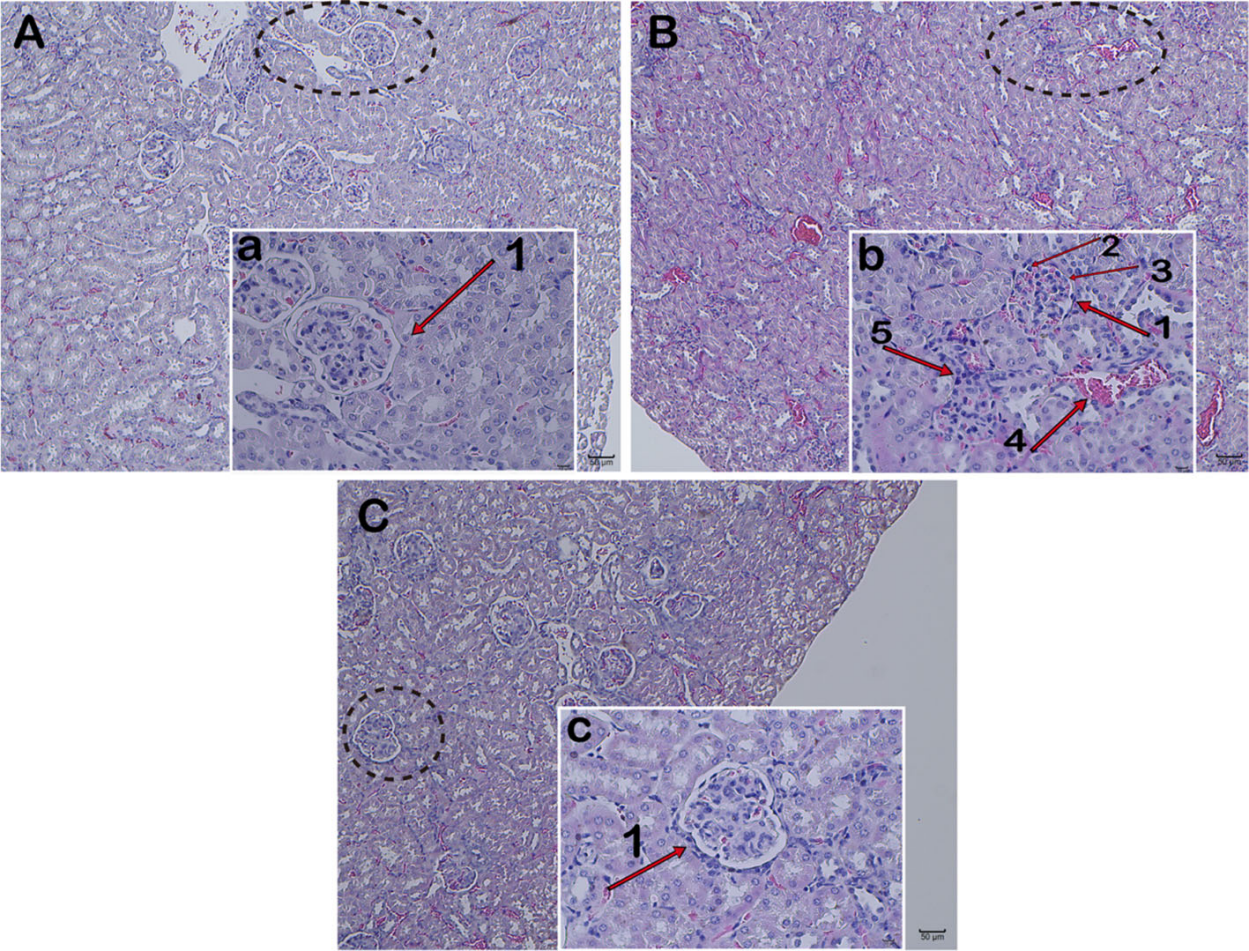
Pathology of kidney tissue. Representative histopathology from each group of MRL/lpr mice: Control group **(A)**, glomerulus in the control group (a), Model group **(B)**, glomerulus in the model group (b), JP group **(C)**, and glomerulus in the JP group (c). Glomerulus (1), glomerular mesangial cells (2), glomerular mesangial matrix (3), renal interstitium (4), and inflammatory cells (5). The control group is MRL/MP mice, and the model group is MRL/MP mice. Magnification (**A–C** are 10×, a, b, c are 40×).

Observation of kidney changes in mice by H&E Staining. Under light microscopy, the model group showed glomerular swelling, mesangial cell proliferation, mesangial matrix hyperplasia, renal interstitial vasodilatation and hyperemia, and a large number of inflammatory cell infiltration (**Figures 5B, b**). Compared with the model group, the proliferation of glomerular mesangial cells in the JP group was declined, and the infiltration of inflammatory cells was alleviated (**Figures 5C, c**). While the renal structure was relatively intact, and there was no hyperplasia and inflammatory cell infiltration in the control group (**Figures 5A, a**). These results suggested that JP could relieve kidney damage.

### JP Can Reduce Urinary Albumin and Anti-dsDNA in Serum of MRL/lpr Mice

**Figure 6:**
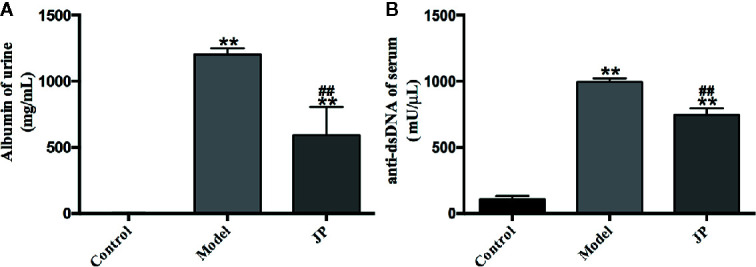
Levels of albumin in urine and levels of anti-dsDNA in serum. These parameters included albumin **(A)** and anti-dsDNA **(B)**. The control group is MRL/MP mice, and the model group is MRL/MP mice. Data are expressed as mean ± SD (n = 6). **P < 0.01 *versus* Control group; ^##^ *P* < 0.01 *versus* Model group.

To elucidate the therapeutic effect of JP, the concentrations of urinary albumin and serum anti-dsDNA were evaluated by ELISA assay. The results of this research illustrated that urinary albumin dramatically decreased in lupus mice after JP intervention. Simultaneously, the serum anti-dsDNA level of the JP group markedly reduced **(** **Figure 6**).

### JP Inhibits the Expressions of Inflammatory Cytokines in BMDMs, Spleen, and Kidney of MRL/lpr Mice

**Figure 7:**
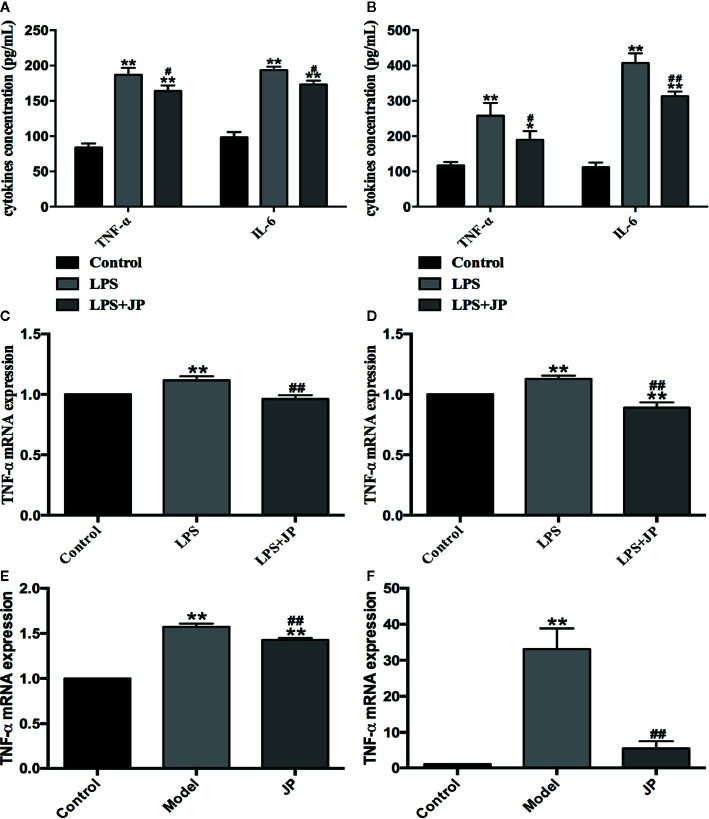
Effect of JP on the expressions of inflammatory cytokines in BMDMs, spleen, and kidney of MRL/lpr mice. Cells were treated with 1 μg/ml LPS either alone or with 2.5% (v/v) JP-treated serum for 24 h. Concentrations of TNF-α and IL-6 in the supernatant of BMDMs in MRL/MP **(A)** and MRL/lpr mice **(B)**. The expression alteration in TNF-α mRNA. BMDMs of MRL/MP **(C)**, BMDMs of MRL/lpr mice **(D)**, spleen of MRL/lpr **(E)**, and kidney of MRL/lpr **(F)**. The control group is MRL/MP mice, and the model group is MRL/MP mice. Data are expressed as mean ± SD (n = 3). *P < 0.05 and **P < 0.01 *versus* Control group; ^#^ *P* < 0.05 and ^##^ *P*< 0.01 *versus* LPS or Model group.

Firstly, we used ELISA to analyze the levels of TNF-α and IL-6 in BMDMs after drug intervention. Under the stimulation of LPS, the secretion of TNF-α and IL-6 in BMDMs increased rapidly. In contrast, the levels of TNF-α and IL-6 were significantly reduced after JP intervention. Meanwhile, these results illustrated that this trend was more pronounced in MRL/lpr than MRL/MP (**Figures 7A, B**). In addition, we analyzed the changes of TNF-α mRNA in BMDMs by JP. Consistent with the ELISA results, the expression of TNF-α mRNA in BMDMs increased dramatically under LPS stimulation. However, the level of TNF-α mRNA was evidently declined in JP-treated cells, especially in the MRL/lpr mice (**Figures 7C, D**).

To further explain this phenomenon, we then discussed it *in vivo*. As expected, the model group showed higher TNF-α mRNA expression in spleen and kidney of MRL/lpr mice. Besides, we detected an obvious decrease in the level of TNF-α mRNA in JP group, especially in kidney (**Figures 7E, F**).

### Effect of JP on Expressions of IRAK1-NF-κB Pathway in BMDMs

**Figure 8:**
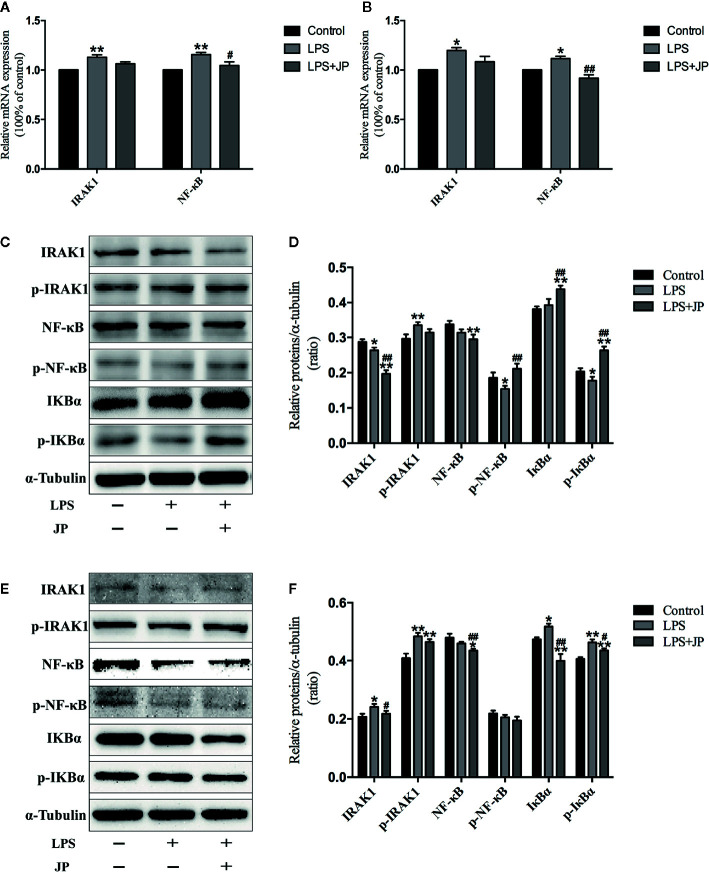
Effect of JP-treated serum on the expressions of IARK1-NF-κB pathway in BMDMs of MRL/MP and MRL/lpr mice. Cells were treated with 1 μg/ml LPS either alone or with 2.5% (v/v) JP-treated serum for 24 h. The mRNA expressions of IARK1 and NF-κB in MRL/MP mice **(A)**. The mRNA expressions of IARK1 and NF-κB in MRL/lpr mice **(B)**. The protein bands of IARK1, p-IARK1, NF-κB, p-NF-κB, IκBα, and p-IκBα in MRL/MP mice **(C)**. Quantitative analysis of protein expression alteration in MRL/MP mice **(D)**. The protein bands of IARK1, p-IARK1, NF-κB, p-NF-κB, IκBα, and p-IκBα in MRL/lpr mice **(E)**. Quantitative analysis of protein expression alteration in MRL/lpr mice **(F)**. Data are expressed as mean ± SD (n = 3). *P < 0.05 and **P < 0.01 *versus* Control group; ^#^ *P* < 0.05 and ^##^ *P* < 0.01 *versus* LPS group.

To further understand the role of IRAK1 molecule in the inflammatory pathway, we performed Western Blot and Rt-qPCR experiments to evaluate IRAK1-NF-κB expression levels based on IRAK1 and NF-κB gene expression in BMDMs. As shown in **Figure 8A**, there was no obvious change in IRAK1 and NF-κB mRNA expression after treated with JP-treated serum in BMDMs of MRL/MP. These implied that JP-treated serum could not affect MRL/MP mice.

Similarly, the protein expressions of IARK1, Phospho-IRAK1 (p-IRAK1), IκBα, Phospho-IκBα (p-IkBα), NF-κB, and Phospho-NF-κB (p-NF-κB) had no distinct change with the treatment of JP-treated serum in BMDMs of MRL/MP (**Figures 8C, D**). Nevertheless, JP-treated serum could affect IRAK1-NF-κB pathway in BMDMs of MRL/lpr. The results of genes and proteins explained that JP group significantly reduced the expression of IRAK1-NF-κB pathway, compared with the LPS group (**Figures 8B, E, F**).

### Effect of JP on Expressions of IRAK1-NF-κB Pathway in Spleens

**Figure 9:**
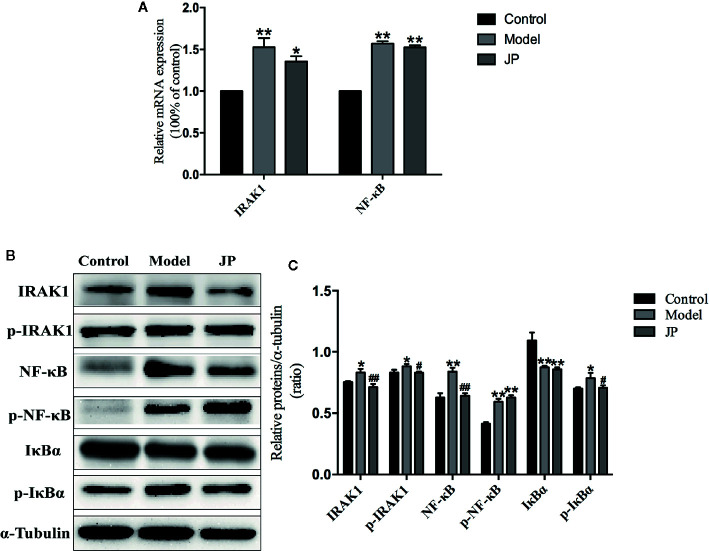
Effect of JP on the expressions of IARK1-NF-κB pathway in spleen of MRL/lpr mice. The mRNA expressions of IARK1 and NF-κB in spleen **(A)**. The protein bands of IARK1, p-IARK1, NF-κB, p-NF-κB, IκBα, and p-IκBα in spleen **(B)**. The protein expression alteration in IARK1, p-IARK1, NF-κB, p-NF-κB, IκBα, and p-IκBα **(C)**. The control group is MRL/MP mice, and the model group is MRL/MP mice. Data are expressed as mean ± SD (n = 3). *P < 0.05 and **P < 0.01 *versus* Control group; ^#^ *P* < 0.05 and ^##^ *P* < 0.01 *versus* Model group.

The IRAK1-NF-κB pathway plays a significant part in regulating the inflammatory activity of autoimmune diseases. Compared with the control group, expressions of IRAK1, p-IRAK1, NF-κB, and p-NF-κB protein were higher in the model group without treatment, which were followed with an increased ratio (**Figures 9B, C**). Increased expressions of IRAK1, p-IRAK1, NF-κB, and p-NF-κB were decreased by administration of JP. Additionally, the Rt-qPCR results also demonstrated that JP lessened the expression of IRAK1 and NF-κB inflammatory signaling pathway genes (**Figure 9A**).

IκBα is the main downstream protein between the IRAK1-NF-κB (Ghosh and Hayden, 2012). Increased expressions of IκBα and p-IκBα were found in spleen of mice from the model group. Nevertheless, IκBα and p-IκBα protein expressions were inhibited by the treatment with JP (**Figures 9B, C**). As mentioned above, it should be illustrated that JP did not lead to a particularly significant reduction in the IRAK1-NF-κB pathway in spleen.

### Effect of JP on Expressions of IRAK1-NF-κB Pathway in Kidneys

**Figure 10:**
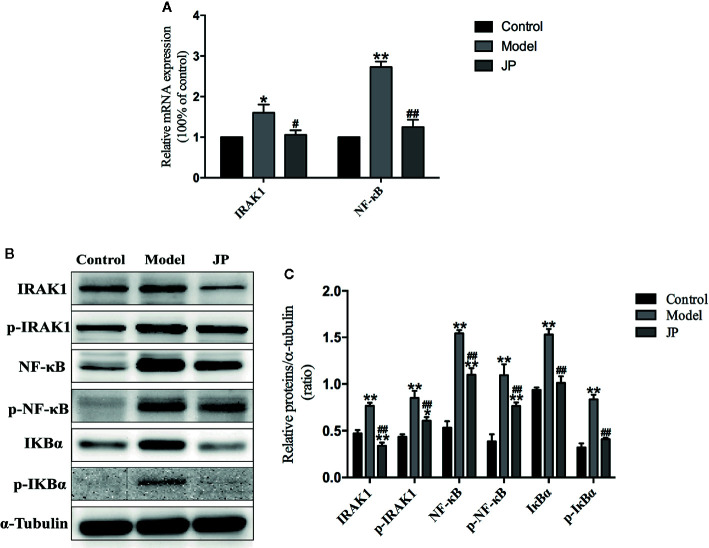
Effect of JP on the expressions of IARK1-NF-κB pathway in kidney of MRL/lpr mice. The mRNA expressions of IARK1 and NF-κB in kidney **(A)**. The protein bands of IARK1, p-IARK1, NF-κB, p-NF-κB, IκBα, and p-IκBα in kidney **(B)**. The protein expression alteration in IARK1, p-IARK1, NF-κB, p-NF-κB, IκBα, and p-IκBα **(C)**. The control group is MRL/MP mice, and the model group is MRL/MP mice. Data are expressed as mean ± SD (n = 3). *P < 0.05 and **P < 0.01 *versus* Control group; ^#^ *P* < 0.05 and ^##^ *P* < 0.01 *versus* Model group.

To evaluate the effect of JP administered on MRL/lpr mice, Rt-qPCR and Western Blot test in kidney were required at the end of the experiment to confirm expressions of IARK1, p-IARK1, IκBα, p-IκBα, NF-κB, and p-NF-κB. The expression levels of IARK1 and NF-κB mRNA in the kidney of the model group remarkably increased, whereas significantly decline were observed in the kidney treated with JP (**Figure 10**).

Interestingly, combining the above renal pathological results, we found that the kidney damage in the model group was more serious. At the same time, the protein expressions of IARK1, p-IARK1, IκBα, p-IκBα, NF-κB, and p-NF-κB in the model group obviously increased. However, the mice of the JP group had less effect on kidney damage with the treatment of JP, and the corresponding IARK1-NF-κB protein expression was significantly reduced (**Figures 10B, C**). Based on the findings above, it suggested that JP could improve the inflammatory damage of lupus kidney by suppressing the IARK1-NF-κB signaling pathway. Accordingly, JP has a therapeutic effect on lupus kidney inflammation in general.

## Discussion

In this research, the effects of JP on the inflammatory response of MRL/lpr mice were investigated *in vivo* and *in vitro*. Compared with BMDMs in MRL/MP, JP significantly reduced the activation of IRAK1 and its downstream inflammatory signals in MRL/lpr mice. What’s more interesting was that JP-treated lupus mice had more significant improvement not only in kidney injury, inflammatory factor secretion but also in the pathway. These new findings support the hypothesis that JP has a role in reducing inflammatory responses and disease progression in SLE. Despite the exact mechanisms have not been fully elucidated, it seems that the IRAK1-NF-κB signaling pathway is a new target for SLE therapy.

Several previous studies have described the therapeutic effects of JP in SLE. *In* *vitro* experiments, JP drug-containing serum inhibited the B cell-activating factor (BAFF) signaling pathway to enhance the role of GC in treating SLE (Wu et al., 2015b). Moreover, JP-treated serum played a synergistic and attenuating role in treating SLE by regulating the TLR9 pathway (Wu et al., 2015a). Besides, we previously investigated the effects of JP on the level of methylation in T lymphocytes to explore the pathogenesis of SLE (Li et al., 2018b). *In vivo* experiments, published studies have found that JP might up-regulate the methylation level of methyl CpG binding protein 2 (MeCP2) in CD4^+^ cells of MRL/lpr mice (Li et al., 2018a). JP is a complex system consisting of a variety of active ingredients such as paeoniflorin and ferulic acid, both of which were discovered in JP and its medicated serum in our study (**Figures 1** and **2**). Paeoniflorin has the function of regulating immune cells, reducing the role of inflammatory mediators, and restoring abnormal signaling pathways (Ji et al., 2018; Zhang and Wei, 2020). As a major active component of JP, ferulic acid has been reported to prevent cell damage resulted from oxidative stress or inflammation, and protect renal damage due to hyperglycemia by regulating autophagy (Chen et al., 2019; Chowdhury et al., 2019). Ferulic acid is also used to regulate the activities of NF-κB in an *in vitro* inflammation model (Lampiasi and Montana, 2018). Additionally, we have separately detected the active ingredients of gallic acid and isoferulic acid in JP (**Figure 1**), gallic acid can protect inflammation and oxidative stress by inhibiting MAPK/NF-κB pathway and may be effective in treating inflammation-related diseases (Tanaka et al., 2018). Isoferulic acid, like ferulic acid, is an active ingredient in Cimicifuga, which has anti-inflammatory and antioxidant properties, and also helps to prevent pancreatic beta-cell dysfunction and apoptosis (Jairajpuri and Jairajpuri, 2016; Meeprom et al., 2018). JP has been shown to be useful in the therapy of SLE, but further elucidation of its mechanism remains valuable.

At present, female MRL/lpr mice are recognized internationally as the most classic SLE model animals, and their symptoms of autoimmune diseases are very similar to those of humans (Fu et al., 2019). For example, MRL/lpr mice are susceptible to glomerulonephritis and other symptoms of kidney damage due to immune dysfunction during growth. Meanwhile, urine protein and blood urea nitrogen (BUN) content increased significantly, and plenty of autoantibodies emerged, such as anti-dsDNA, anti-ssDNA, anti-nuclear antibodies (ANA), etc. As expected in the present study, MRL/lpr mice in the model group demonstrated significant renal pathological changes, markedly increased urine albumin levels, and increased anti-dsDNA levels in serum (**Figures 5B** and **6A, B**). Macrophage plays an antigen presentation function, inflammation regulation and immune induction functions. As a pattern recognition receptor, TLR mainly exists on the surface of macrophages, especially TLR4. And TLR4 is the main source of inflammatory cytokines in the body, and its expression is related to a diversity of inflammatory responses (Zhang et al., 2013). Additionally, LPS is crucial in the pathogenesis of SLE, and the main receptor that can recognize LPS on the surface of macrophages is TLR4 (Han et al., 2018). The abnormal function of macrophages may destroy the immune homeostasis of the human body and induce autoimmune diseases simultaneously (Zhang et al., 2010). Moreover, recent research has proved that macrophage-associated surface molecules in SLE patients had abnormal immune regulation and phagocytosis, and targeting macrophages might be a new strategy for treating SLE (Chen et al., 2018). We found in previous experiments that mouse peritoneal macrophages were obtained in smaller numbers. Thus, in this experiment, we isolated BMDMs of MRL/MP and MRL/lpr lupus mice as research objects and explored the association between the SLE and IRAK1 pathways at the cellular and molecular level. As expected, the stimulation of mouse BMDMs by LPS increased the higher expressions of inflammatory cytokines and IRAK1-NF-κB inflammatory signaling pathways in MRL/lpr than MRL/MP. After JP intervention, the secretions of inflammatory cytokines in BMDMs were reduced, which illustrated that JP effectively inhibited IRAK1 and its downstream NF-κB inflammation signaling pathways (**Figures 7A, B** and **8**).

SLE is a typical autoimmune disease with multiple clinical manifestations. The immune complex is deposited on the blood vessels of the kidney and spleen, which can recruit complement factors, leading to inflammation and organ pathological damage (Nanda et al., 2011; Chen et al., 2012; Zhou et al., 2018). Among them, renal involvement is a severe complication with poor prognosis, and if not treated, it will result in pathological changes of renal parenchyma and the replacement of fibrotic tissue (Yung and Chan, 2015). These are consistent with our experimental results. The kidney tissue of the control group mice was normal, while the tissue of the model group implied typical characteristics of nephritis, such as infiltration of inflammatory cells and significant hyperplasia of thin membrane matrix. However, symptoms in the JP group were alleviated (**Figure 5**). Studies have unveiled the promoted levels of TNF-α and C-C Motif Chemokine Ligand 5 (CCL5) mRNA provoked by TLR4 and TLR7 in spleen macrophages of lupus-susceptible mice (Murphy et al., 2017). The spleen of mice in the model group also showed pathological conditions, such as splenomegaly (Zhang et al., 2019). Additionally, the expression levels of IRAK1-NF-κB in the kidneys of the model group were higher than those in the spleen, and the effects of JP intervention were also significantly reduced in the kidneys (**Figures 7E, F**, **9**, **10**). It was reported that NF-κB up-regulated pro-inflammatory mediators to promote the infiltration of macrophages into renal tissue, and then induced the progression of lupus nephritis (Hu et al., 2015; Liu et al., 2015). It has been shown that macrophages are critical in the development of lupus, especially in kidney inflammation (Triantafyllopoulou et al., 2010; Chalmers et al., 2015; Li et al., 2015). Therefore, we speculate that the kidney is more suitable as a research object in the IRAK1-NF-κB pathway in this article, but the precise mechanism of action needs further verification.

**Figure 11:**
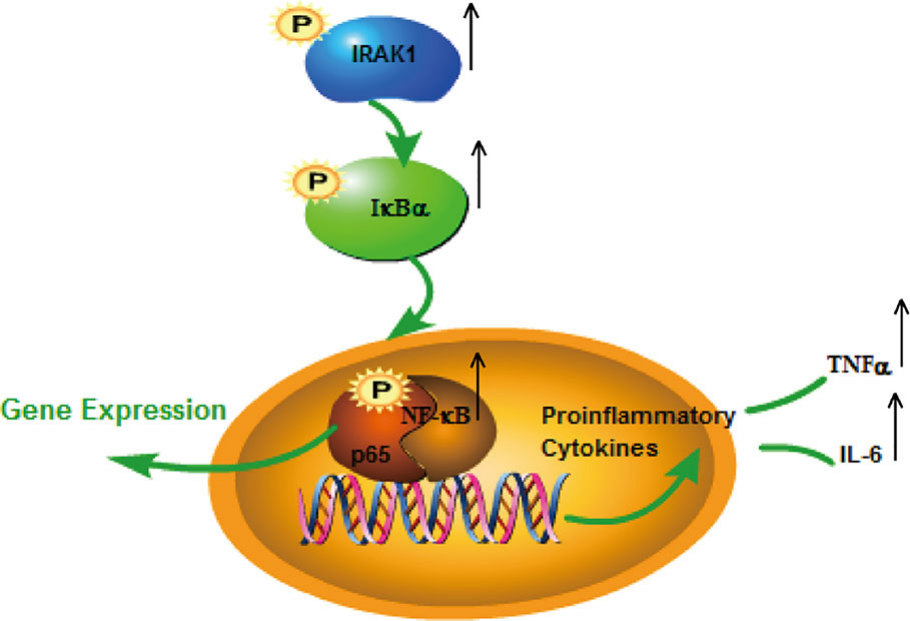
Possible molecular mechanisms associated with SLE: IRAK1-NF-kappaB pathway.

The role of IRAK1 molecular targets in the pathogenesis of SLE has attracted attention for the past few years. We previously explored the role of IRAK1 in the NF-κB related pathway from lupus model mice through *in vitro* experiments (Ji et al., 2018). And this article is to investigate the mechanism of IRAK1 in lupus. Consequently, we speculated that the suppression of IRAK1 activity might inhibit inflammation in mice models of lupus. IRAK1 is related to the abnormal activation of NF-κB signal during the pathogenesis of SLE (**Figure 11**). The study revealed that the typical NF-κB protein bounds to IκB which is phosphorylated by activating the IKK complex, allowing translocation of the NF-κB protein into the nucleus. Specific DNA sequences can interact with activated NF-κB to induce target proteins and eventually mediate inflammatory responses (Ghosh and Hayden, 2012). In the present study, the expressions of IκB/p-IκB mRNA and protein in the spleen, kidney, as well as LPS-induced BMDMs of MRL/lpr mice in the model group were dramatically increased after JP intervention, which was consistent with our assumption. Moreover, the occurrence and development of SLE have relations with enhanced inflammatory response and over-activated pro-inflammatory cytokines (MA et al., 2011). The expressions of TNF-α and IL-6 in lupus patients are generally higher than that in healthy people, and studies have reported that the lack of IRAK1 could reduce the production of IL-6 and TNF-α (Uematsu et al., 2005; Willis et al., 2012). The results showed that JP could inhibit inflammatory cytokines in both cell and animal experiments. Therefore, these data further confirmed that JP had anti-inflammatory activity (**Figure 7**), suggesting that the anti-inflammatory ability of JP in lupus might be partially achieved through the IRAK1-NF-κB signaling.

Overall, research data demonstrated that JP had a role in reducing inflammatory activity in SLE by inhibiting IRAK1-NF-κB signaling activity in MRL/lpr mice and their BMDMs. All these findings provide another way for JP to learn more about the mechanism and principle of JP in clinical treatment of SLE.

## Data Availability Statement

The original contributions presented in the study are included in the article/**Supplementary Files**, further inquiries can be directed to the corresponding author/s.

## Ethics Statement

All animal experiments were reviewed and approved by the Committee on the Ethics of Animal Experiments of Zhejiang Chinese Medical University and adhered to the guidelines of the Guide for the Care and Use of Laboratory Animals.

## Author Contributions

LJ and XF performed the experiments and wrote the paper. XH provided technical guidance. DF, JB, AZ, and SC participated in the preliminary experimental preparation and analyzed the data. RL and YF secured the funding and revised the manuscript. All authors contributed to the article and approved the submitted version.

## Funding

This work was supported by the National Natural Science Foundation of China (No. 81673863), the National Natural Science Foundation of Zhejiang Province (Nos. LY19H290006, LQ18H270004, and LY18H290007), the Youth Program of National Natural Science Foundation of China (No. 81803980), 2020 Annual Scientific Research Fund of Zhejiang Chinese Medical University (No. 2020ZR06), and Zhejiang First-Class Discipline (Chinese Medicine) Open Fund.

## Conflict of Interest

The authors declare that the research was conducted in the absence of any commercial or financial relationships that could be construed as a potential conflict of interest.
