# Supplementary figures and images for: Jieduquyuziyin Prescription Suppresses Inflammatory Activity of MRL/lpr Mice and Their Bone Marrow-Derived Macrophages via Inhibiting Expression of IRAK1-NF-κB Signaling Pathway
Source: Front Pharmacol. 2020 Jul 14;11:1049. doi: 10.3389/fphar.2020.01049 (PMC7372094; doi:10.3389/fphar.2020.01049)

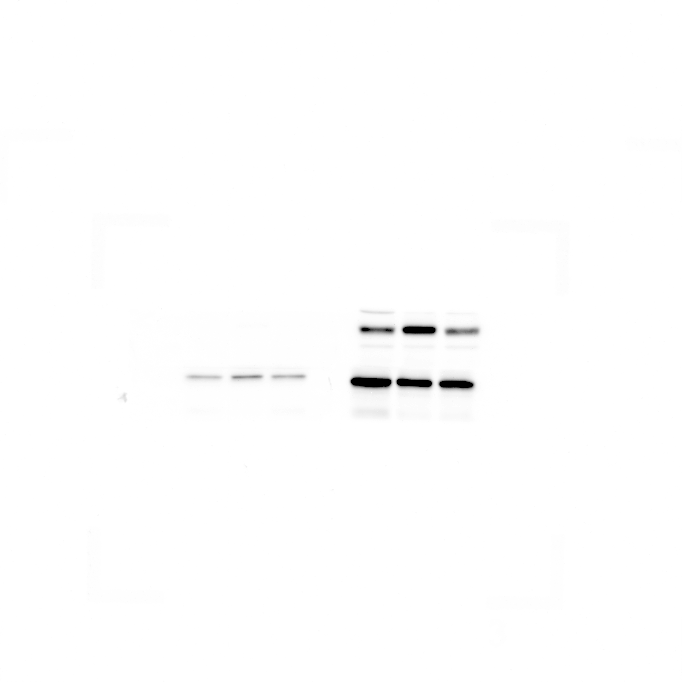

Supplement: Supplementary file 1 [file DataSheet_1.zip › Original protein data/Kidney/IkB.tif]

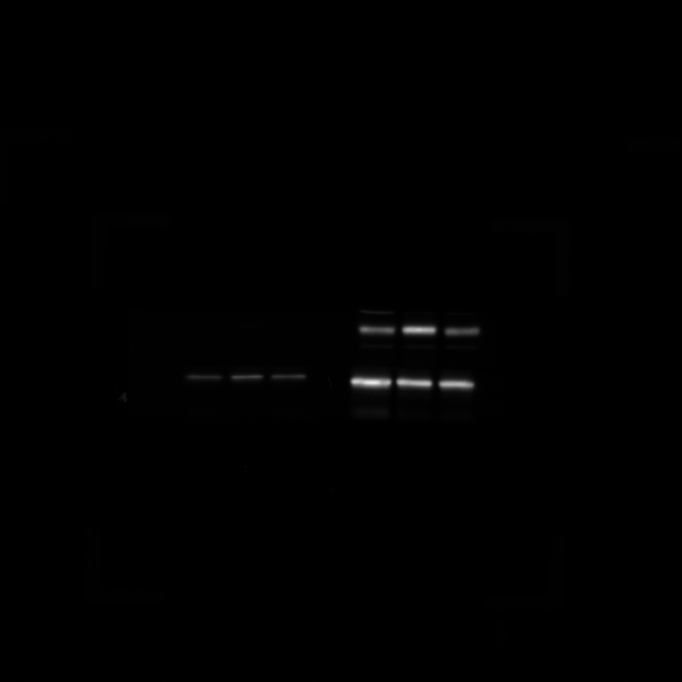

Supplement: Supplementary file 1 [file DataSheet_1.zip › Original protein data/Kidney/Kidney-Ikb-original.tif]

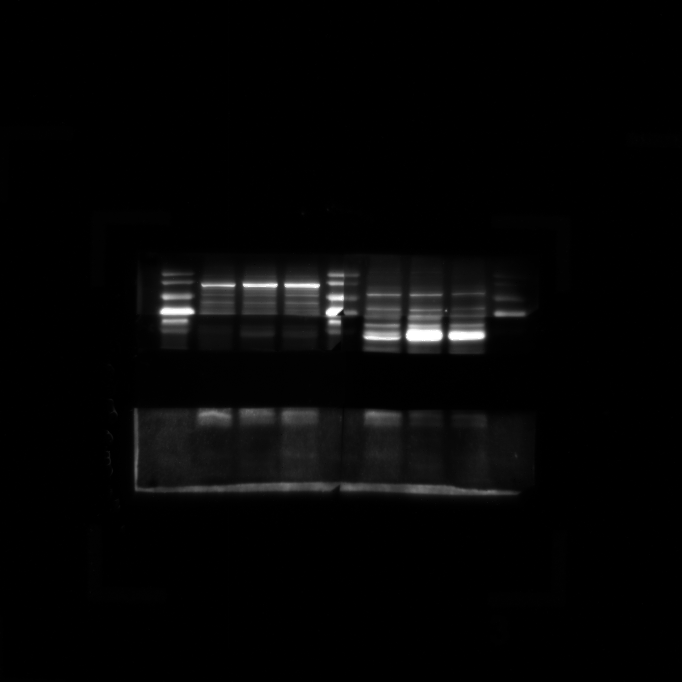

Supplement: Supplementary file 1 [file DataSheet_1.zip › Original protein data/Kidney/Kidney-IRAK1-original.tif]

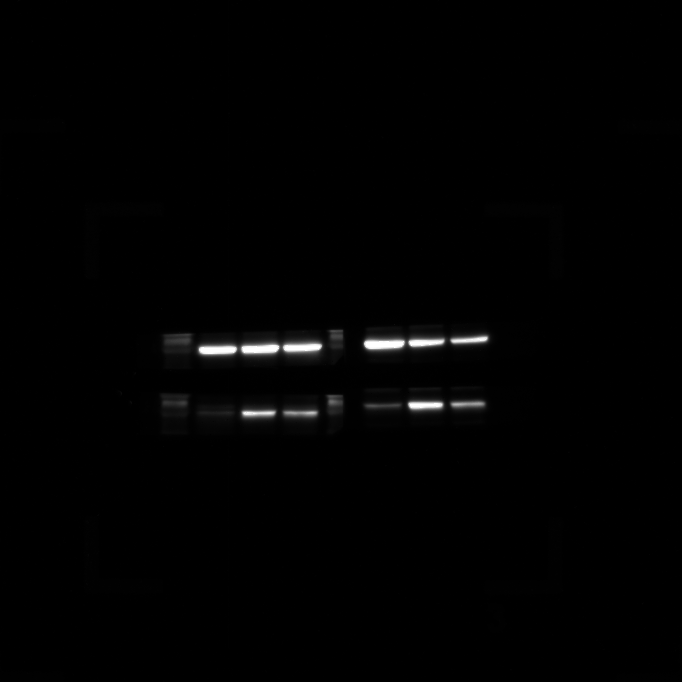

Supplement: Supplementary file 1 [file DataSheet_1.zip › Original protein data/Kidney/Kidney-tubulin-original.tif]

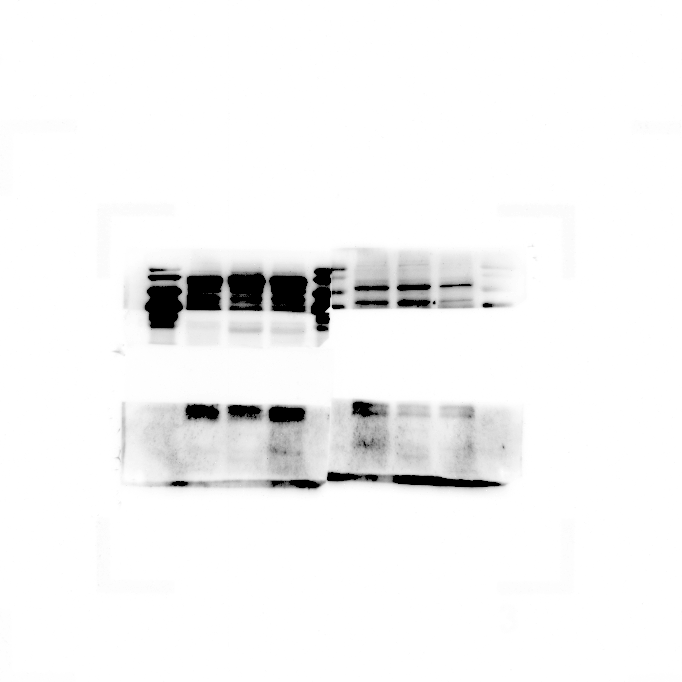

Supplement: Supplementary file 1 [file DataSheet_1.zip › Original protein data/Kidney/lRAK1.tif]

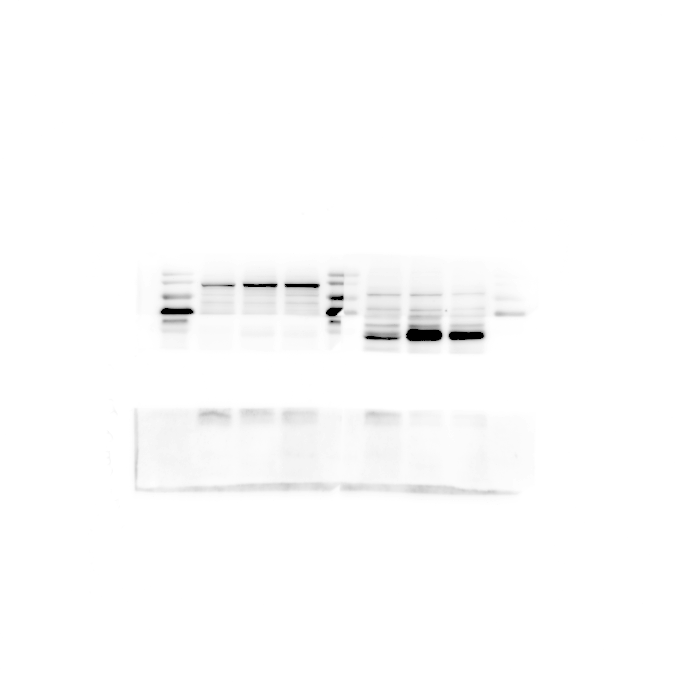

Supplement: Supplementary file 1 [file DataSheet_1.zip › Original protein data/Kidney/NF-kB.tif]

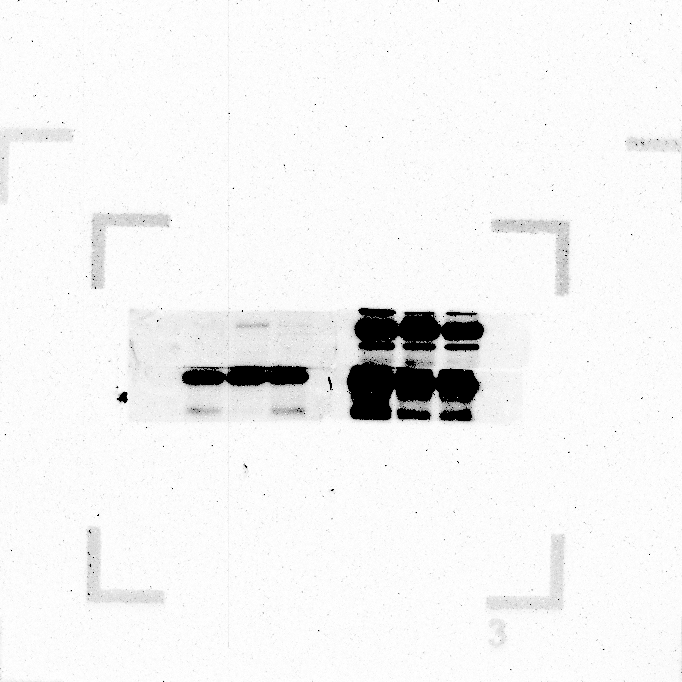

Supplement: Supplementary file 1 [file DataSheet_1.zip › Original protein data/Kidney/p-Ikb.tif]

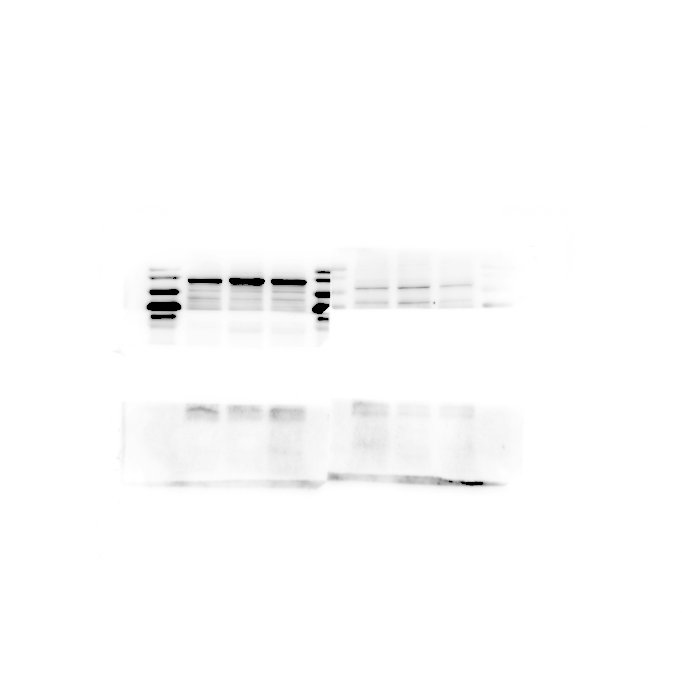

Supplement: Supplementary file 1 [file DataSheet_1.zip › Original protein data/Kidney/p-lRAK1.tif]

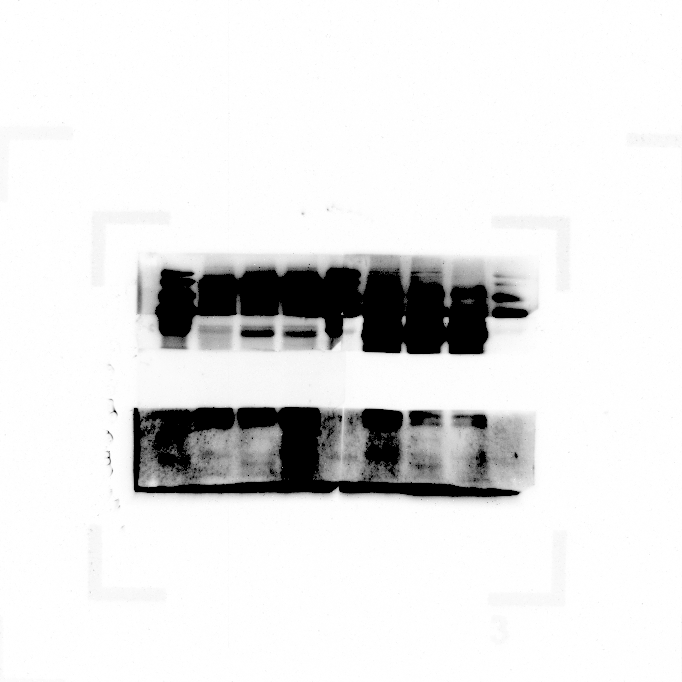

Supplement: Supplementary file 1 [file DataSheet_1.zip › Original protein data/Kidney/p-NF-kB.tif]

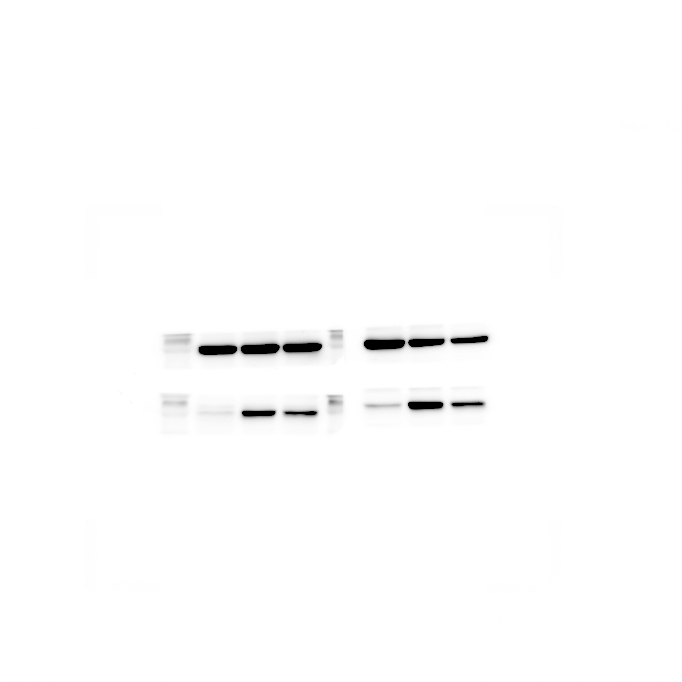

Supplement: Supplementary file 1 [file DataSheet_1.zip › Original protein data/Kidney/tubulin.tif]

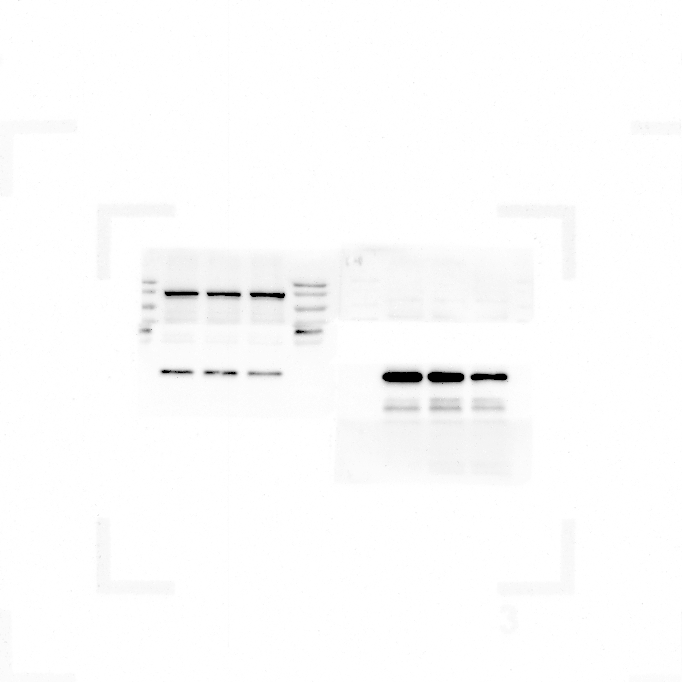

Supplement: Supplementary file 1 [file DataSheet_1.zip › Original protein data/MRL:lpr BMDMs/IkB.tif]

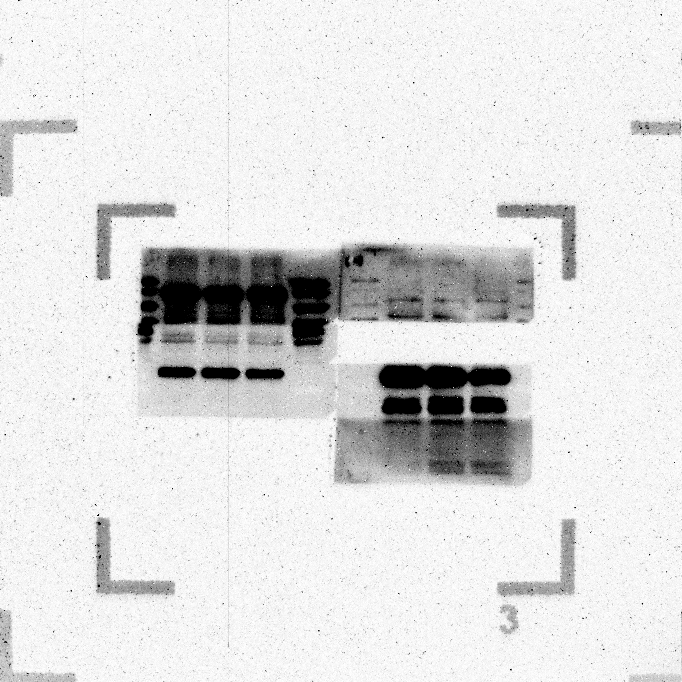

Supplement: Supplementary file 1 [file DataSheet_1.zip › Original protein data/MRL:lpr BMDMs/IRAK1.tif]

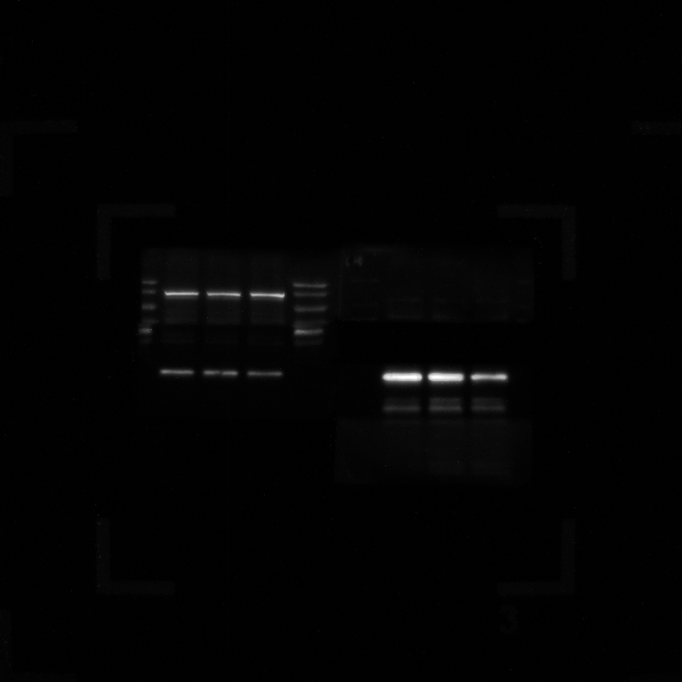

Supplement: Supplementary file 1 [file DataSheet_1.zip › Original protein data/MRL:lpr BMDMs/MRL:lpr BMDMs-IkB-original.tif]

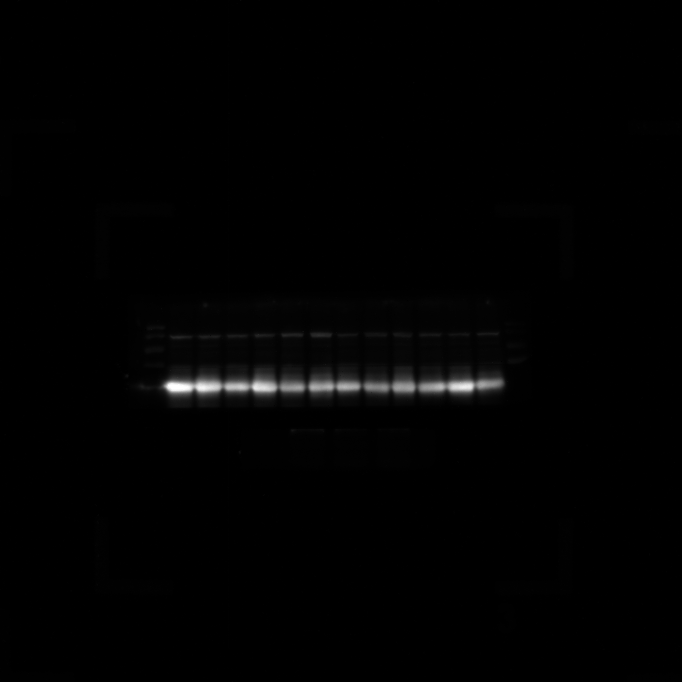

Supplement: Supplementary file 1 [file DataSheet_1.zip › Original protein data/MRL:lpr BMDMs/MRL:lpr BMDMs-NF-kB-original.tif]

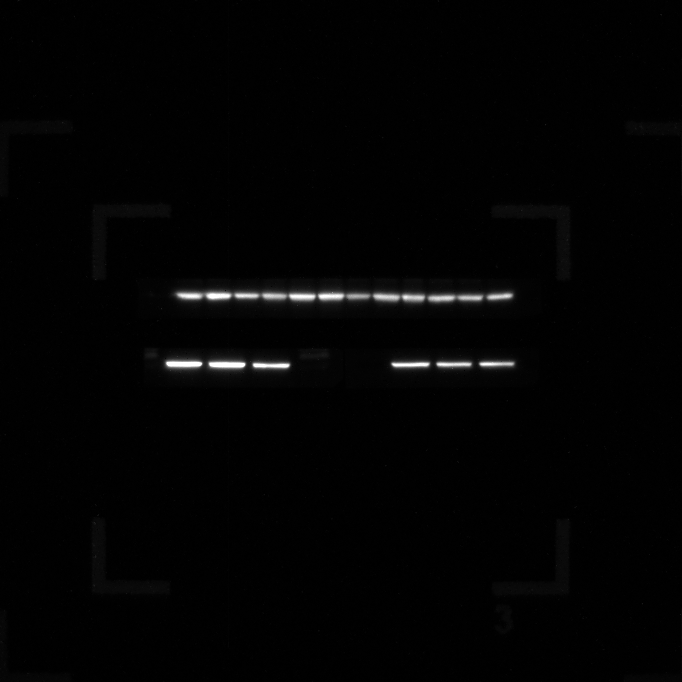

Supplement: Supplementary file 1 [file DataSheet_1.zip › Original protein data/MRL:lpr BMDMs/MRL:lpr BMDMs-tubulin-original.tif]

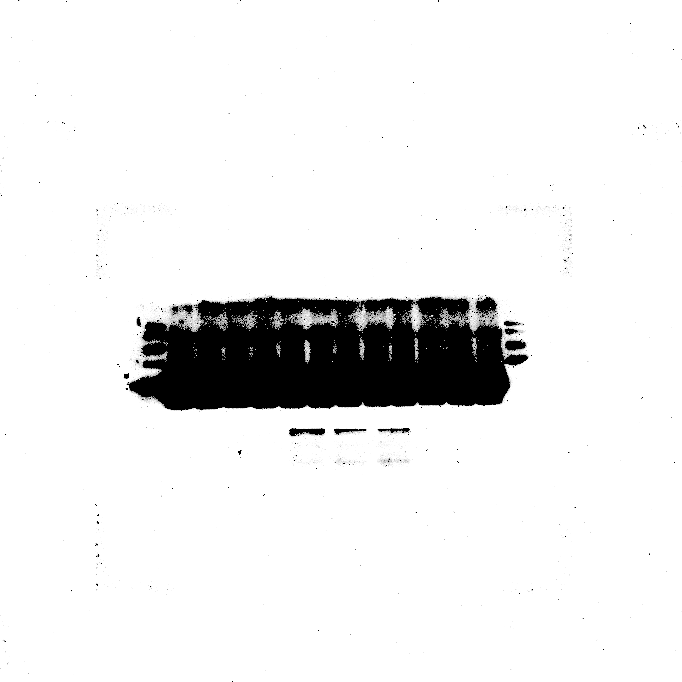

Supplement: Supplementary file 1 [file DataSheet_1.zip › Original protein data/MRL:lpr BMDMs/NF-kB.tif]

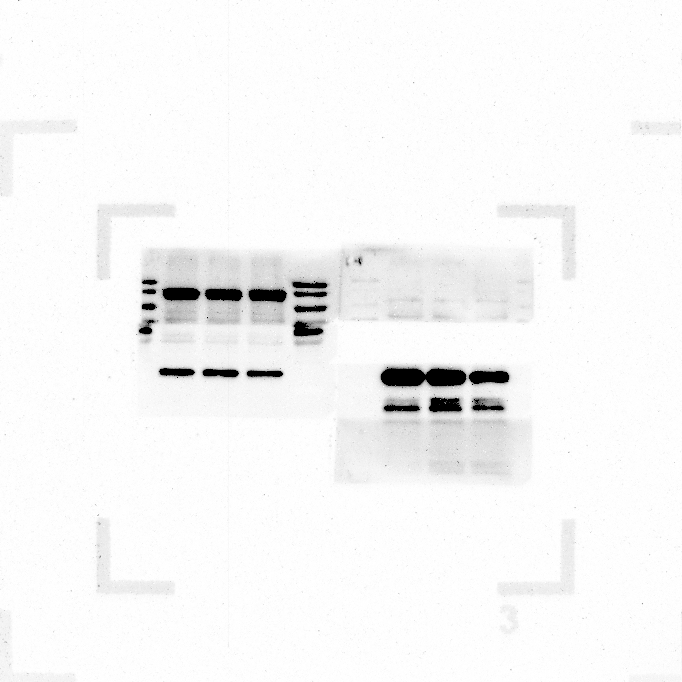

Supplement: Supplementary file 1 [file DataSheet_1.zip › Original protein data/MRL:lpr BMDMs/p-IkB.tif]

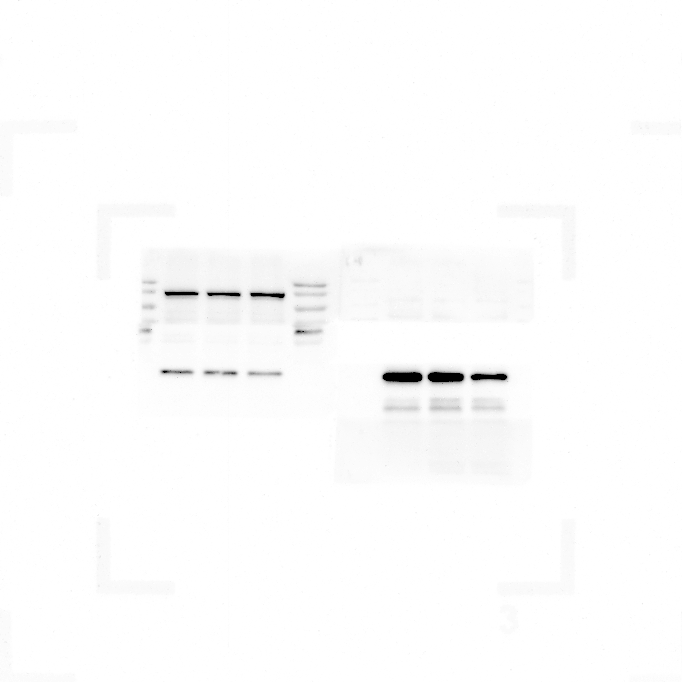

Supplement: Supplementary file 1 [file DataSheet_1.zip › Original protein data/MRL:lpr BMDMs/p-lRAK1.tif]

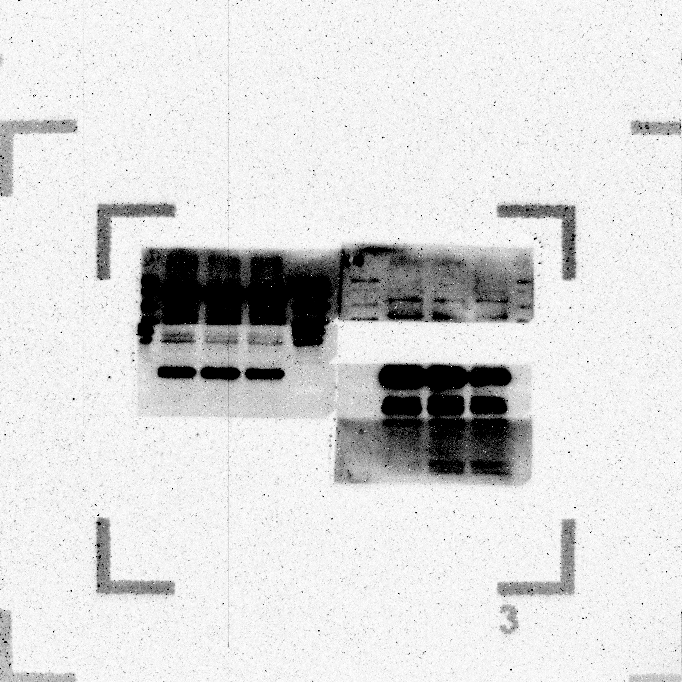

Supplement: Supplementary file 1 [file DataSheet_1.zip › Original protein data/MRL:lpr BMDMs/p-NF-kB.tif]

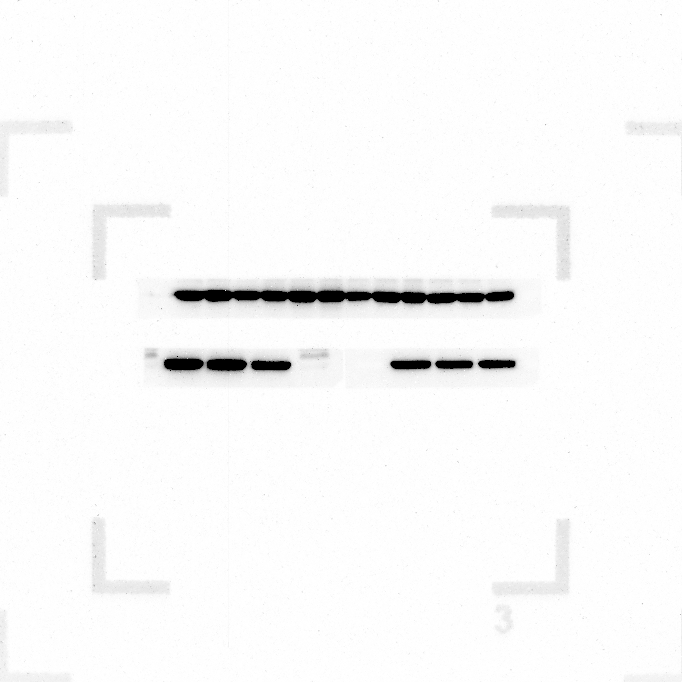

Supplement: Supplementary file 1 [file DataSheet_1.zip › Original protein data/MRL:lpr BMDMs/tubulin.tif]

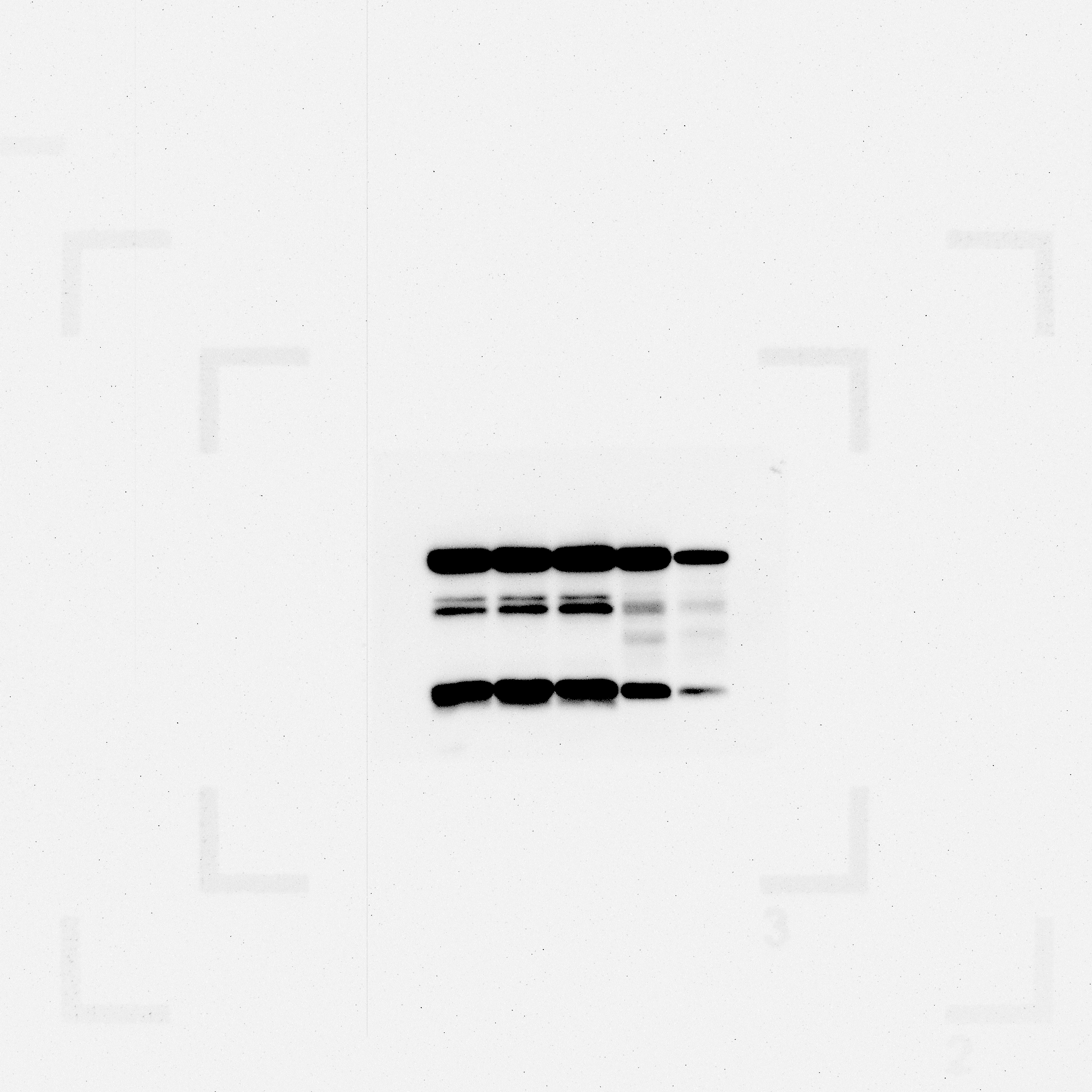

Supplement: Supplementary file 1 [file DataSheet_1.zip › Original protein data/MRL:MP BMDMs/IkB.tif]

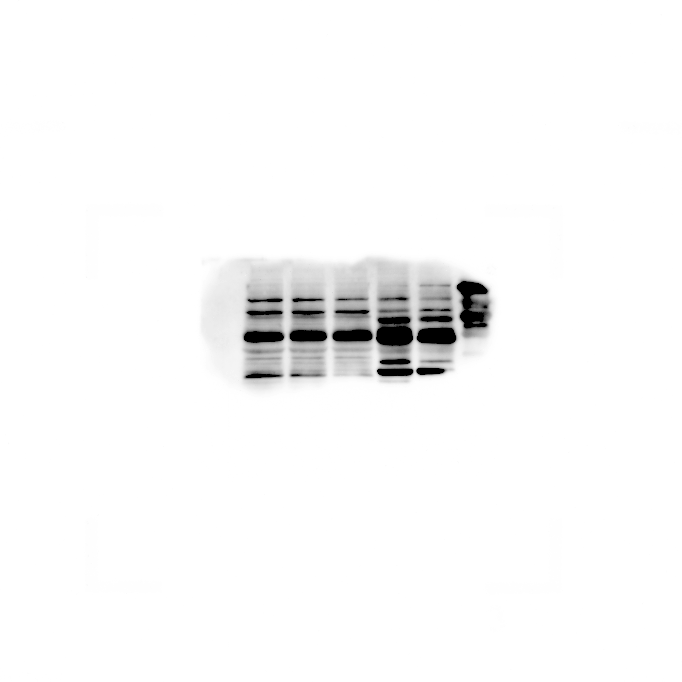

Supplement: Supplementary file 1 [file DataSheet_1.zip › Original protein data/MRL:MP BMDMs/IRAK1.tif]

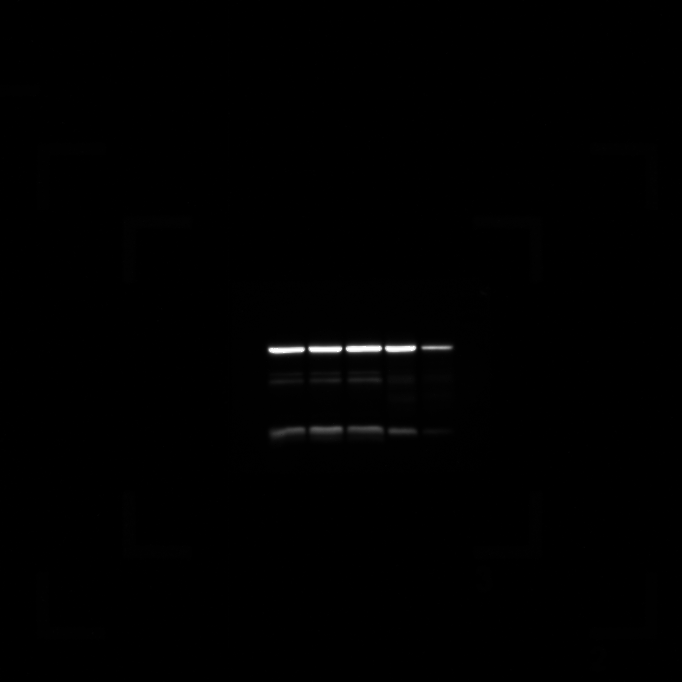

Supplement: Supplementary file 1 [file DataSheet_1.zip › Original protein data/MRL:MP BMDMs/MRL:MP BMDMs-IkB-orginal.tif]

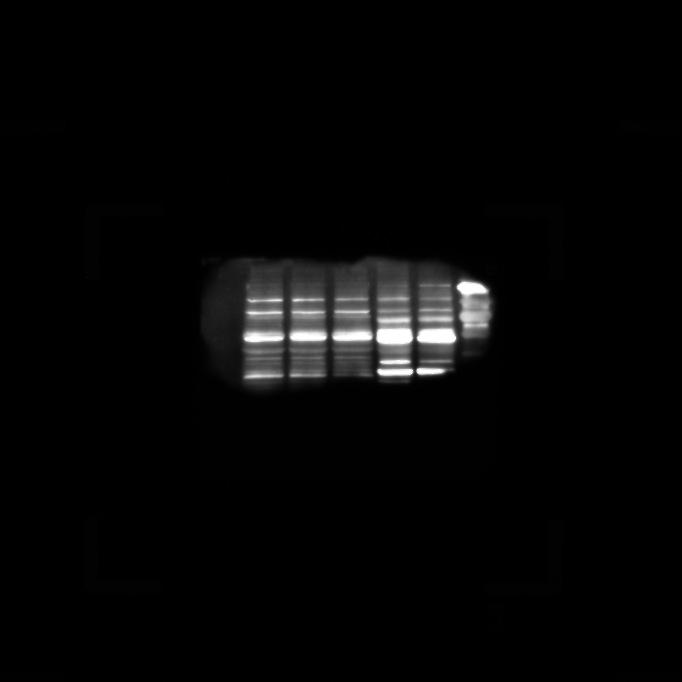

Supplement: Supplementary file 1 [file DataSheet_1.zip › Original protein data/MRL:MP BMDMs/MRL:MP BMDMs-IRAK1-original.tif]

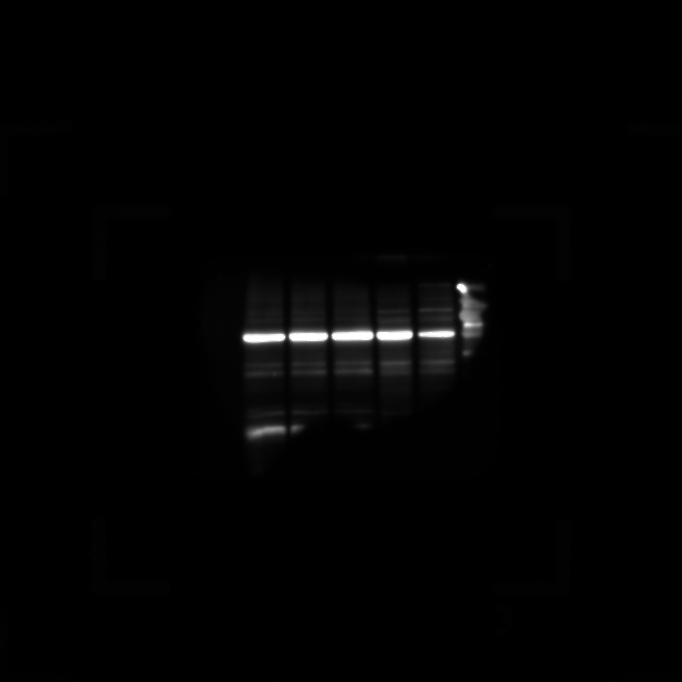

Supplement: Supplementary file 1 [file DataSheet_1.zip › Original protein data/MRL:MP BMDMs/MRL:MP BMDMs-NF-kB-original.tif]

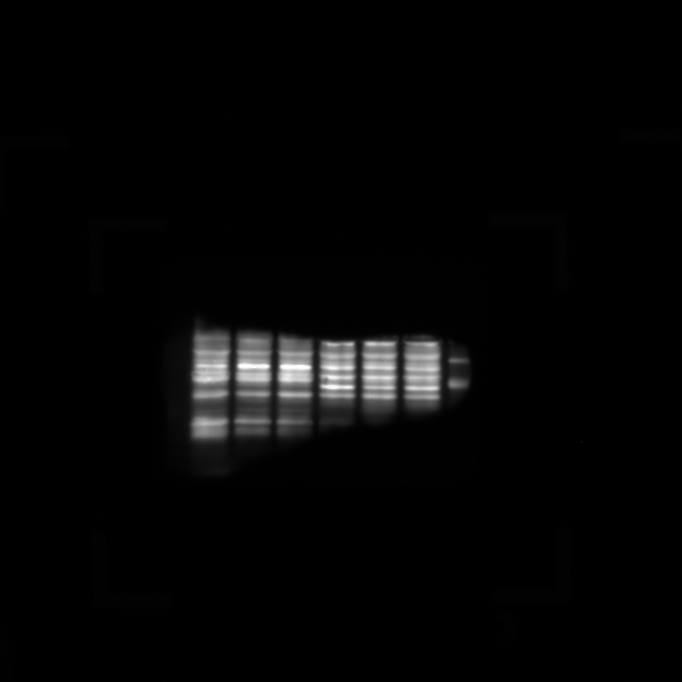

Supplement: Supplementary file 1 [file DataSheet_1.zip › Original protein data/MRL:MP BMDMs/MRL:MP BMDMs-p-IkB-original.tif]

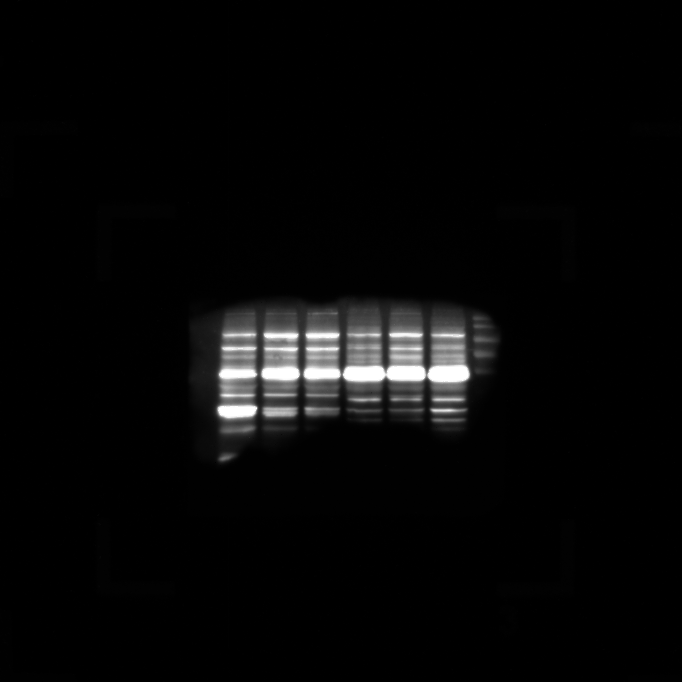

Supplement: Supplementary file 1 [file DataSheet_1.zip › Original protein data/MRL:MP BMDMs/MRL:MP BMDMs-p-IRAK1-original.tif]

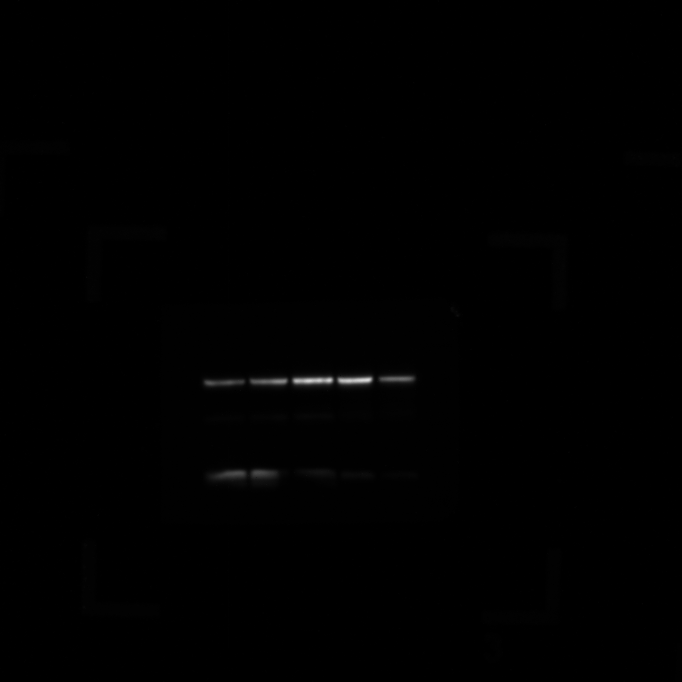

Supplement: Supplementary file 1 [file DataSheet_1.zip › Original protein data/MRL:MP BMDMs/MRL:MP BMDMs-tubulin-original.tif]

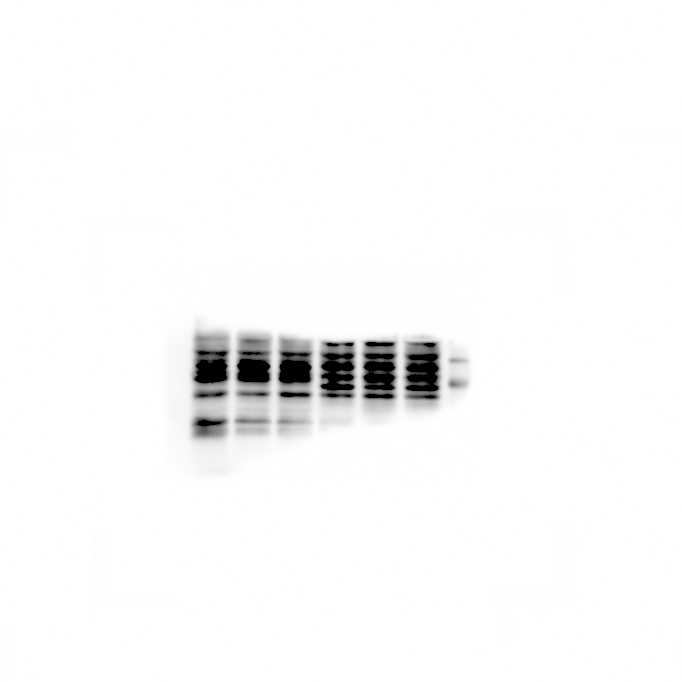

Supplement: Supplementary file 1 [file DataSheet_1.zip › Original protein data/MRL:MP BMDMs/p-IkB.tif]

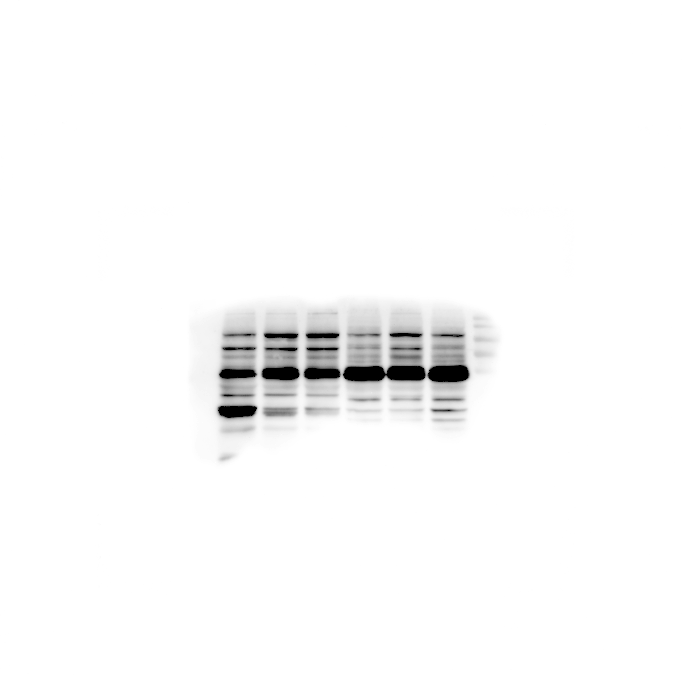

Supplement: Supplementary file 1 [file DataSheet_1.zip › Original protein data/MRL:MP BMDMs/p-IRAK1.tif]

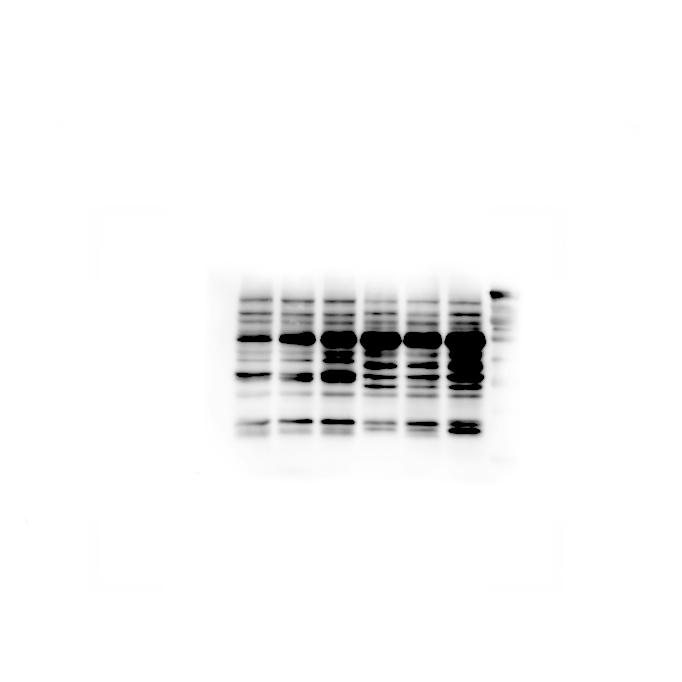

Supplement: Supplementary file 1 [file DataSheet_1.zip › Original protein data/MRL:MP BMDMs/p-NF-kB.tif]

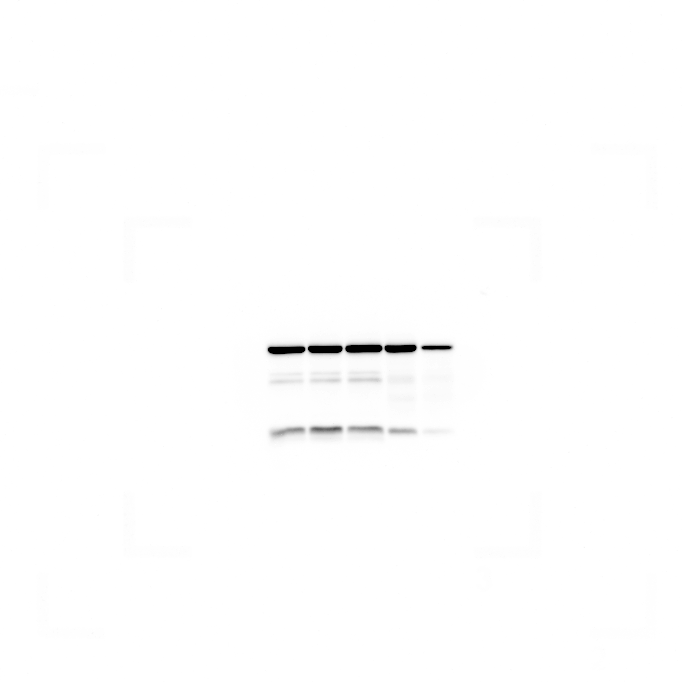

Supplement: Supplementary file 1 [file DataSheet_1.zip › Original protein data/MRL:MP BMDMs/tubulin.tif]

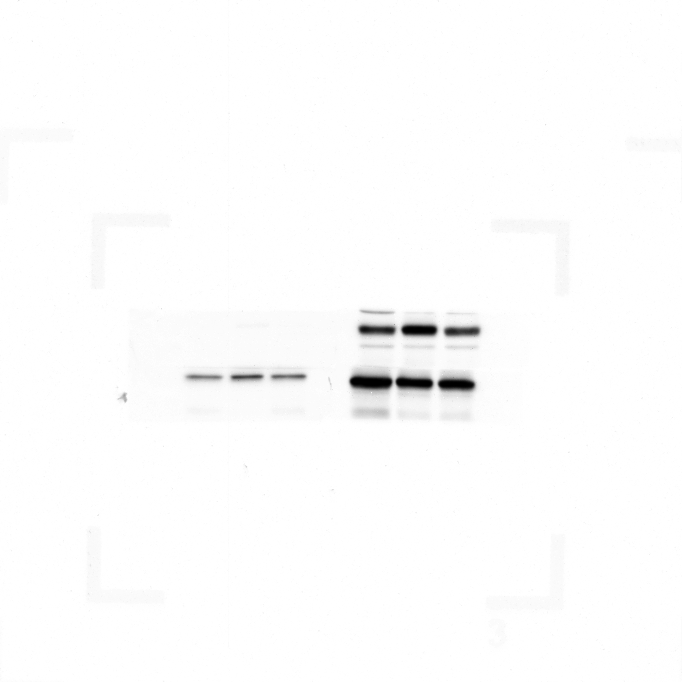

Supplement: Supplementary file 1 [file DataSheet_1.zip › Original protein data/Spleen/IkB.tif]

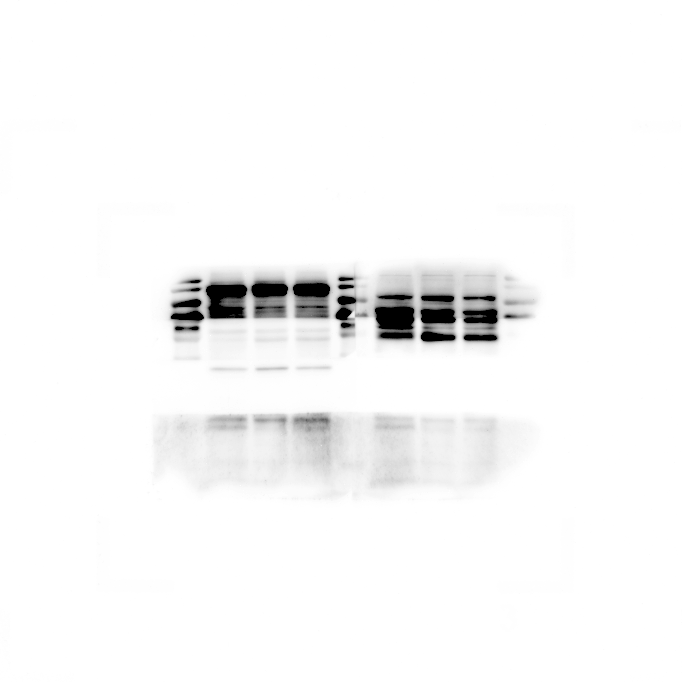

Supplement: Supplementary file 1 [file DataSheet_1.zip › Original protein data/Spleen/IRAK1.tif]

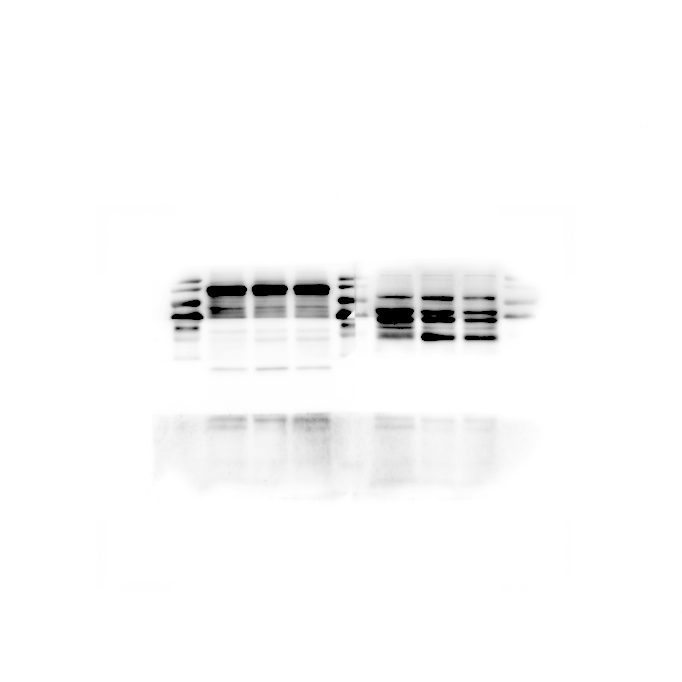

Supplement: Supplementary file 1 [file DataSheet_1.zip › Original protein data/Spleen/NF-kB.tif]

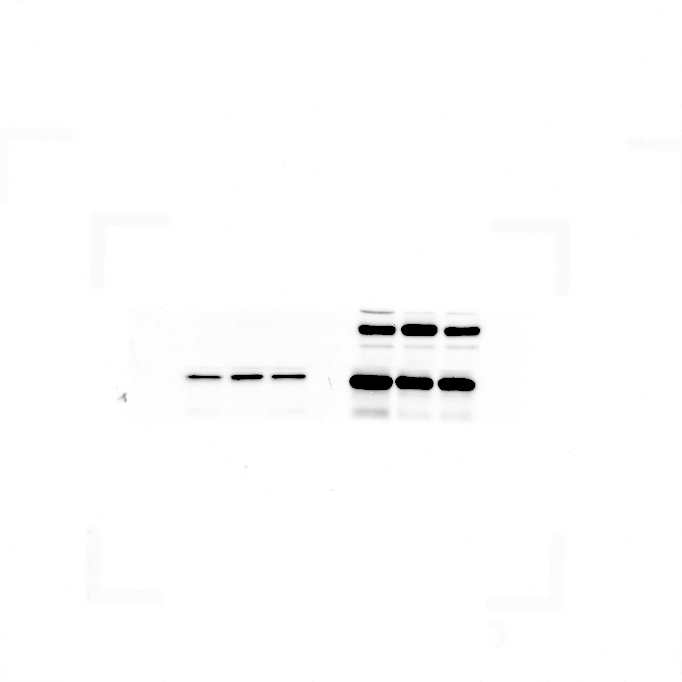

Supplement: Supplementary file 1 [file DataSheet_1.zip › Original protein data/Spleen/p-IkB.tif]

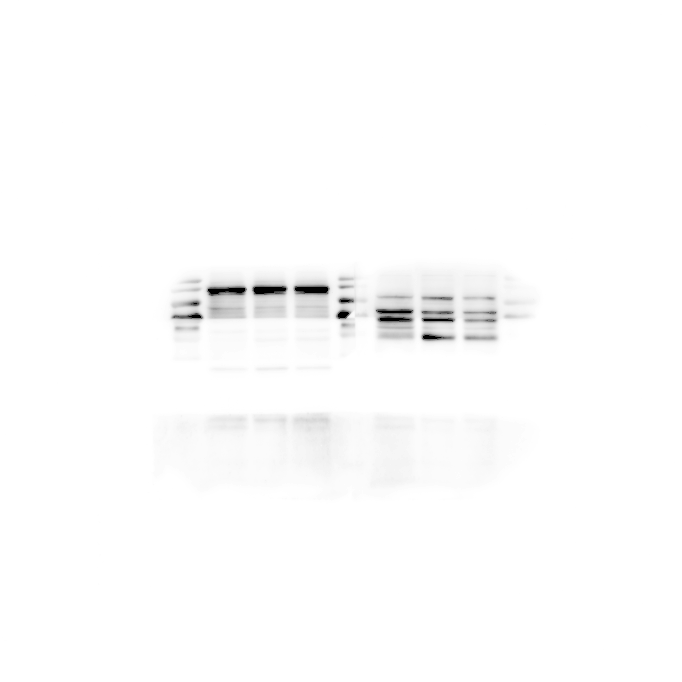

Supplement: Supplementary file 1 [file DataSheet_1.zip › Original protein data/Spleen/p-lRKA1.tif]

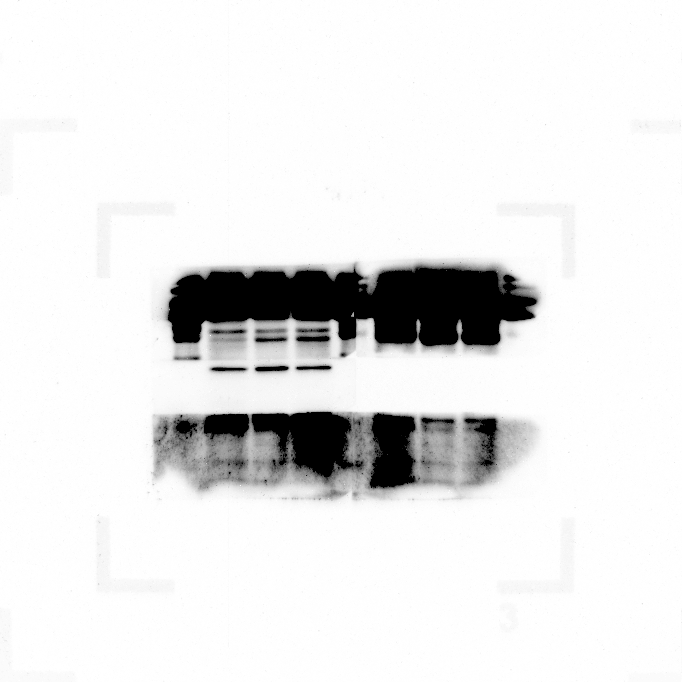

Supplement: Supplementary file 1 [file DataSheet_1.zip › Original protein data/Spleen/p-NF-kB.tif]

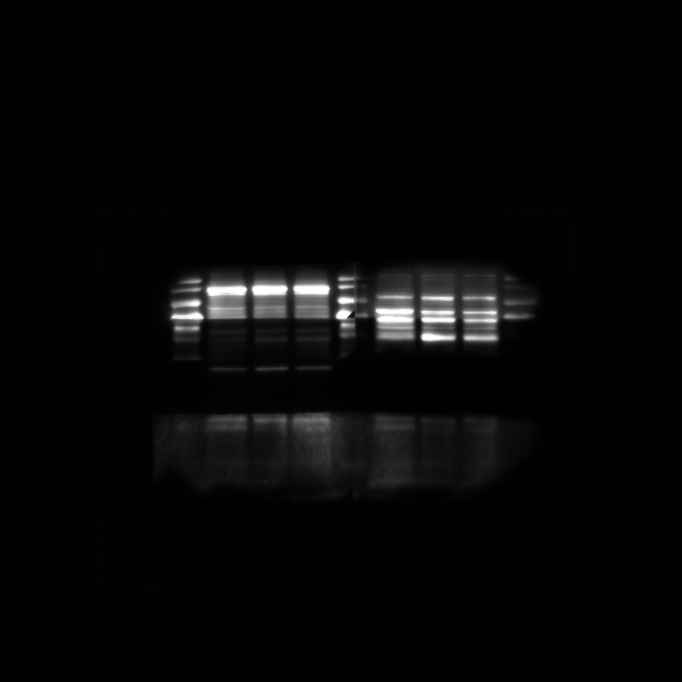

Supplement: Supplementary file 1 [file DataSheet_1.zip › Original protein data/Spleen/Spleen-IRAK1-original.tif]
